# Astrocytes Control Circadian Timekeeping in the Suprachiasmatic Nucleus via Glutamatergic Signaling

**DOI:** 10.1016/j.neuron.2017.02.030

**Published:** 2017-03-22

**Authors:** Marco Brancaccio, Andrew P. Patton, Johanna E. Chesham, Elizabeth S. Maywood, Michael H. Hastings

**Affiliations:** 1 Division of Neurobiology, MRC Laboratory of Molecular Biology, Cambridge CB2 0QH, UK

**Keywords:** astrocytic-neuronal interactions, calcium oscillations, membrane potential oscillations, circadian, SCN, extracellular glutamate, NMDAR2C, GABA, circuit synchronization, circadian behavior

## Abstract

The suprachiasmatic nucleus (SCN) of the hypothalamus orchestrates daily rhythms of physiology and behavior in mammals. Its circadian (∼24 hr) oscillations of gene expression and electrical activity are generated intrinsically and can persist indefinitely in temporal isolation. This robust and resilient timekeeping is generally regarded as a product of the intrinsic connectivity of its neurons. Here we show that neurons constitute only one “half” of the SCN clock, the one metabolically active during circadian daytime. In contrast, SCN astrocytes are active during circadian nighttime, when they suppress the activity of SCN neurons by regulating extracellular glutamate levels. This glutamatergic gliotransmission is sensed by neurons of the dorsal SCN via specific pre-synaptic NMDA receptor assemblies containing NR2C subunits. Remarkably, somatic genetic re-programming of intracellular clocks in SCN astrocytes was capable of remodeling circadian behavioral rhythms in adult mice. Thus, SCN circuit-level timekeeping arises from interdependent and mutually supportive astrocytic-neuronal signaling.

## Highlights

• SCN neurons are active during circadian day, but SCN astrocytes are active at night
• Astrocytes direct circadian cycles of extracellular glutamate to inhibit SCN neurons
• Astrocyte-derived inhibition is mediated by NMDAR2C complexes on dorsal SCN neurons
• Genetic re-programming of the clock in SCN astrocytes reshapes circadian behavior

## Teaser

Neurons of the suprachiasmatic nucleus (SCN) are responsible for circadian pacemaking in mammals. Here, Brancaccio et al. demonstrate that SCN astrocytes also possess pacemaking properties and unravel how they set circadian tempo by reciprocal timing with their neuronal partners.

## Introduction

The suprachiasmatic nucleus (SCN), the master circadian clock in mammals, coordinates daily rhythms of behavior and metabolism (Reppert and Weaver, 2002). Circadian time (CT) is defined by cell-autonomous, transcription-translational feedback loops (TTFLs), in which expression of the *Period* and *Cryptochrome* genes is suppressed by their protein products (Hastings et al., 2014). Comparable TTFLs exist in most tissues across the body, but they require SCN-derived periodic input to ensure robustness and coherence of internal circadian programs. In contrast, the SCN is uniquely able to sustain persistent circadian molecular and electrophysiological oscillations ex vivo (Brancaccio et al., 2014). This robust pacemaking is widely viewed as a product of neuropeptidergic inter-neuronal signaling across the SCN circuit (Liu et al., 2007, Maywood et al., 2011). Recently, the roles of different neuronal subpopulations in the SCN have been assessed by selective genetic manipulations (see Herzog et al., 2017). Collectively, these data indicate that neurons in the dorsal SCN, mainly expressing arginine-vasopressin (AVP), are pacemaker cells, capable of imposing their intrinsic periodicity to mouse behavior, whereas neurons expressing vasoactive intestinal peptide (VIP) in the ventral region are important for light entrainment and internal synchronization. Nevertheless, the molecular, cellular, and circuit properties that specifically characterize the dorsal and ventral SCN are obscure. Much attention has been given to different neuronal SCN subpopulations, but the role of SCN astrocytes in encoding CT has been largely overlooked, although a role in the *Drosophila* clock has been indicated (Jackson, 2011). SCN astrocytes express high levels of glial fibrillary acidic protein (GFAP), which exhibits 24 hr oscillations in its distribution, both in light-dark conditions and in constant darkness (Lavialle and Servière, 1993, Santos et al., 2005). Any active contribution of SCN astrocytes to circadian pacemaking is, however, currently undetermined. Cortical astrocytes exhibit circadian oscillations, but these rhythms disappear after a week in cell culture, suggesting a more passive role in this brain area (Prolo et al., 2005). Critically, however, dispersed cell culture generally depletes glial cultures of the associated neurons and destroys the cyto-architecture of the associated neuronal circuits. This profoundly alters the micro-environmental conditions in which astrocytic function is normally exerted, thereby potentially confounding interpretations.

To determine whether astrocytes play an active role in circadian timekeeping, we combined in vivo studies in mice with ex vivo analysis of circadian properties of SCN organotypic slices, in which the integrity of the glial and neuronal counterparts is preserved (Brancaccio et al., 2013). By using long-term live imaging, we simultaneously co-detected circadian oscillations of neuronal and astrocytic [Ca^2+^]_i_ within the SCN and found them to be anti-phasic. By using different metabolic markers, we confirmed that astrocytes are active during the circadian night, whereby they release glutamate in the extracellular space to inhibit neuronal activation in the SCN. Pharmacological interference with astrocytically released glutamate, or inhibition of specific subunits of the NMDA receptors (NMDARs) (NR2C) expressed in the dorsal SCN, suppressed electrical and molecular circadian oscillations within the nucleus and desynchronized its neuronal circuit, thus showing that astrocytes are necessary for circadian timekeeping. We reconstruct a novel circuit model for the SCN, featuring a dorsal astrocytic-neuronal anti-phasic oscillatory microcircuit, responsible for circadian timekeeping in mammals. To test this model, we manipulated the intrinsic period of the SCN astrocytic TTFL in vivo and found that such treatment altered circadian patterns of locomotor activity, similarly to analogous manipulations delivered to SCN neurons. Thus, SCN astrocytes are not a passive component in circadian pacemaking: they intermesh with, and sculpt, the SCN neural circuit to establish an inter-cellular logical axis that specifies CT.

## Results

### SCN Astrocytes Express Robust Circadian Rhythms of [Ca^2+^]_i_ in Anti-phase to Neuronal [Ca^2+^]_i_ Rhythms

**Figure 1:**
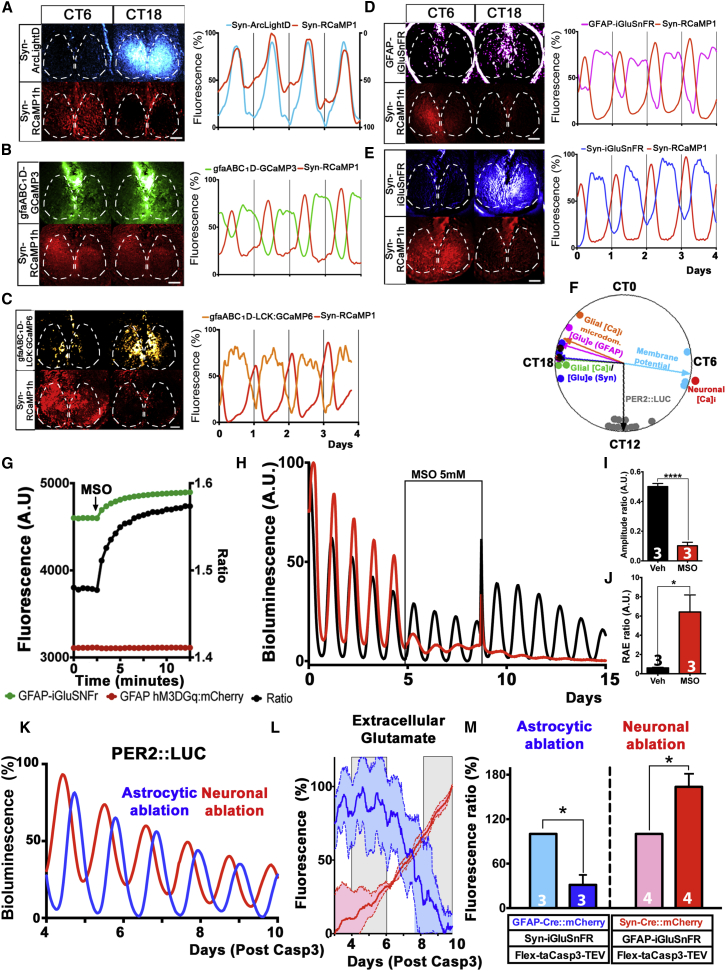
Circadian Landscape of Interlocked, Anti-phasic Oscillations in SCN Neurons and Astrocytes (A–E) Photomicrographs and representative traces of SCN slices transduced with AAVs encoding Syn-RCaMP1h reporting neuronal [Ca^2+^]_i_ paired with (A) Syn-ArcLightD reporting membrane voltage; (B) gfaABC1D-GCaMP3 reporting astrocytic [Ca^2+^]_i_; (C) gfaABC1D-LCK::GCaMP6 detecting [Ca^2+^]_i_ in astrocytic microdomains; and (D and E) iGluSnFR driven by Syn or GFAP, respectively, to detect [Glu]_e_ localized on neuronal or astrocytic cell membranes. ArclightD y axis inverted for presentation purposes. SCN area is outlined (white dashed lines) for presentation purposes. (F) Circadian landscape of reporters in (A)–(E), plotted relative to phase of RCaMP1h (n depicted on graph; each dot is a biological replicate). PER2::LUC is used to register circadian time (CT12). Photomicrographs are false colors LUT ΔF/F. (G) Fluorescent traces and ratios showing GFAP-iGluSnFR fluorescence upon MSO treatment, compared with co-expressed GFAP-hM3DGq::mCherry, used as an internal control. n = 3 for both reporters. (H) Representative traces of circadian oscillations of PER2::LUC in SCN slices treated with MSO or vehicle, in serum-free conditions. (I and J) Bar graphs showing amplitude ratio (I) and relative amplitude error (RAE) ratio (J) in SCN slices treated with MSO or vehicle. (K) Representative traces of PER2::LUC oscillations of SCN slices treated with astrocytically (blue line) or neuronally (red line) restricted flex-taCasp3-TEV to specifically ablate those populations. (L) Mean ± SEM iGluSnFR traces from astrocytically ablated or neuronally ablated SCN slices. Scale bars, 100 μM. Measurement windows in gray. (M) Bar graphs showing variations of iGluSnFR fluorescent intensity ratios in astrocytically or neuronally ablated SCN slices. All bar graphs are mean ± SEM; n experimental replicates depicted on bars. Statistical tests are as follows: (I and J) one-way ANOVA, Bonferroni corrected; (M) two-tailed paired t test. ^∗^p < 0.05; ^∗∗∗∗^p < 0.0001. See also Figures S1–S4 and Movie S1.

Neuronal [Ca^2+^]_i_ shows high-amplitude circadian oscillations in SCN slices, as reported by GCaMP3 driven by the neuronally restricted promoter of human Synapsin 1 (Syn) and delivered ex vivo by adeno-associated viral vectors (AAVs) (Brancaccio et al., 2013). To accommodate other genetically encoded reporters with neuronal [Ca^2+^]_i_ imaging, we validated the use of the red calcium reporter RCaMP1h (Akerboom et al., 2013) in SCN slices. Syn-RCaMP1h fluorescence exhibited high-amplitude circadian oscillations, comparable to those previously reported by Syn-GCaMP3, both in phase and amplitude (Figures S1 and 1A–1E). The peak phase of the RCaMP1h (determined as described previously; Brancaccio et al., 2013) was 6.6 ± 0.2 hr (mean ± SEM, n = 13), similar to what was reported for the Syn-GCaMP3 reporter. To confirm that circadian oscillations of neuronal [Ca^2+^]_i_ are a faithful proxy for neuronal activation in the SCN, we co-transduced SCN slices with AAVs expressing the green voltage indicator Syn-ArcLightD (Cao et al., 2013, Jin et al., 2012) together with Syn-RCaMP1h. Co-detection of the two reporters revealed persistent circadian oscillations of neuronal membrane potential, peaking in mid circadian day in direct register with neuronal [Ca^2+^]_i_ (Figure 1A), thus confirming neuronal [Ca^2+^]_i_ as a bona fide report of daily neuronal activation across the SCN. To assess circadian function in astrocytes, SCN slices were transduced with AAVs encoding GCaMP3 driven by the astrocyte-restricted gfaABC_1_D promoter (Shigetomi et al., 2013). Over a timeframe of seconds, astrocytes exhibited fast, coordinated bursts of [Ca^2+^]_i_ activation (Figure S2), as described in other brain regions (Scemes and Giaume, 2006). More surprisingly, however, they also exhibited marked circadian oscillations of gfaABC_1_D-GCaMP3-reported [Ca^2+^]_i_ (Figure 1B), thus revealing a new layer of complexity to the regulation of astrocytic [Ca^2+^]_i_ (Khakh and Sofroniew, 2015, Volterra et al., 2014). Remarkably, these oscillations of [Ca^2+^]_i_ were anti-phasic to those of neuronal [Ca^2+^]_i_ co-detected in the red channel in the same SCN, and, moreover, they showed a complementary waveform (Figure 1B). Furthermore, selective detection of the Ca^2+^ signal in SCN astrocytic microdomains by AAVs expressing the membrane-tethered gfaABC_1_D-LCK::GCaMP6 (Shigetomi et al., 2010, Shigetomi et al., 2013) confirmed the anti-phasic temporal relationship with neuronal [Ca^2+^]_i_. Interestingly, however, it also revealed a stronger expression in the dorsal SCN, in contrast to the diffused signal detected by the cytosolic [Ca^2+^]_i._ reporter (Figures 1B and 1C). This suggests that although circadian rhythms of [Ca^2+^]_i_ are similarly present in both the soma and microdomains of SCN astrocytes, the dorsal SCN may be a particularly important site for the mediation of astrocytic-neuronal interactions across CT.

### SCN Expresses Circadian Oscillations of Extracellular Glutamate in Phase with Astrocytic [Ca^2+^]_i_

To further examine the nature of such interactions, we focused on extracellular glutamate ([Glu]_e_), a major gliotransmitter in other brain areas (Malarkey and Parpura, 2008), recently implicated in switching cortical circuits to a highly synchronous slow-wave sleep-like state (Poskanzer and Yuste, 2016). The vast majority (>95%) of SCN neurons are GABAergic (Abrahamson and Moore, 2001); thus, the genetically encoded glutamate sensor iGluSnFR (Marvin et al., 2013) will selectively report non-synaptic extracellular glutamate. Syn-iGluSnFR revealed widespread circadian oscillations of [Glu]_e_ in the SCN (Movie S1), and, consistent with its ambient presence in the extracellular space, [Glu]_e_ circadian rhythms were reported equally by iGluSnFR localized either on neurons or on astrocytes (detected by GFAP-iGluSnFR). Moreover, combined use with Syn-RCaMP1h demonstrated that [Glu]_e_ oscillations were anti-phasic to neuronal [Ca^2+^]_i_ and thereby in phase with astrocytic [Ca^2+^]_i_ (Figures 1D and 1E). Overall, therefore, these results showed a sharp temporal segregation of neuronal and astrocytic circadian activities in the SCN, respectively peaking at CT6.5 and CT18.5, with [Glu]_e_ oscillations in tune with astrocytic activation (Figure 1F).

### Astrocytic Control of Extracellular Glutamate Rhythms in SCN

To investigate if [Glu]_e_ originates specifically from astrocytes, as implied by the profile of temporal activation, we pursued three different strategies. First, we confirmed that [Glu]_e_ and PER2::LUC (Yoo et al., 2004) oscillations in the SCN persist indefinitely in medium without exogenous sources of glutamate (serum and glutamine) (Figures S3A and S3B). Thus, circadian oscillations of [Glu]_e_ are generated intrinsically in the SCN, and given that neurons cannot synthesize glutamate de novo, these results support the astrocytic origin of [Glu]_e_ (Schousboe et al., 2013). Second, glutamate is converted to glutamine by the cytosolic activity of glutamine synthase (GS), which is exclusively expressed in astrocytes (Norenberg and Martinez-Hernandez, 1979). Therefore, blocking GS activity by using methionine sulfoximine (MSO) (Weisbrod and Meister, 1973) increases cytosolic glutamate levels in astrocytes, in turn augmenting its release into the extracellular space, presumably via increased vesicular transport (Ni and Parpura, 2009, Perez et al., 2012). As reported by GFAP-iGluSnFR, treating SCN slices with MSO increased [Glu]_e_ within seconds, consistent with the idea that the observed circadian rhythms of [Glu]_e_ also depend directly on astrocytic metabolism (Figure 1G). Moreover, this [Glu]_e_ rise was accompanied by severe damping of circadian PER2::LUC rhythms (Figures 1H–1J), thus showing that control of [Glu]_e_ by astrocytic metabolism is necessary for correct molecular timekeeping in the SCN.

### Selective Targeting and Genetic Manipulation Reveals Glutamate-Mediated Astrocyte-Neuronal Coupling in SCN

To further demonstrate the astrocytic origin of [Glu]_e_, we genetically ablated neurons or astrocytes in the SCN by selectively promoting apoptosis in these cells and observed the effects of this treatment on [Glu]_e_ levels in real time (Figures 1K–1M and S3C–S3F). To do this, we used AAVs to target expression of the Cre recombinase, fused to mCherry, to neurons or astrocytes in SCN slices by using Syn or GFAP promoters, respectively. Consistent and comparable cell numbers were targeted by Syn and GFAP expressing AAVs, as quantified by mCherry::Cre expression; moreover, both promoters delivered similarly high rates of Cre recombination, as estimated by co-transduction with a flexed EF1α-EYFP AAV reporter (>81% for both) (Figures S3C and S3D). This was confirmed when SCN slices were simultaneously transduced with AAVs expressing Cre either by Syn or by GFAP, respectively fused to mCherry and EGFP (Figures S3D and S3E). Importantly, no significant overlapping (∼0.6%) of these reporters was detected in these conditions (Figure S3E), thus confirming the specificity of our targeting to the neuronal and astrocytic populations, respectively. To further demonstrate that GFAP-mCherry::Cre^+^ only targeted astrocytes and not other glial cell types, we also co-immunostained SCN slices expressing GFAP-mCherry::Cre with a panel of markers specific for different glial lineages (Aldh1L1 for astrocytes, NG2 for oligodendrocytes, and Iba1 for microglia). Whereas we observed high levels of mCherry co-localization with the astrocyte-specific marker Aldh1L1, as expected, we found no co-localization with the markers specific for other glial lineages (Figure S3F).

Having confirmed the efficiency and specificity of the GFAP promoter in targeting our genetic reporters and manipulations specifically to SCN astrocytes, we then super-transduced Syn or GFAP-mCherry::Cre^+^ slices with EF1α-flex-taCasp3-TEVp AAVs to specifically promote in these cells apoptosis mediated by a genetically engineered activated form of caspase 3 (Yang et al., 2013). In the early phases of the caspase expression, the astrocytic ablation selectively decreased [Glu]_e_, whereas ablating SCN neurons had the opposite effect, as reported by iGluSnFR fluorescence. Importantly, co-detected PER2::LUC oscillations were sustained in these slices, thus excluding any indirect effect arising from generalized cell death (Figures 1K–1M). Together, our results not only confirmed the astrocytic origin of extracellular glutamate in the SCN, but also highlighted a potential role for SCN neurons in the clearance of glutamate from the extracellular space (see below). As previously mentioned, SCN neurons are GABAergic (Abrahamson and Moore, 2001) and so [Glu]_e_ levels should not be affected by tetrodotoxin (TTX)-mediated blockage of synaptic release in SCN slices. Consistent with this, we detected no variation in glutamate levels in slices treated with high concentrations of TTX (1 μM) (Figures S4A and S4B). Moreover, [Glu]_e_ rhythms persisted in these conditions over several days, albeit with a reduced circadian amplitude (Figure S4C). We interpreted these results as (indirect) evidence of the interdependence of astrocytic and neuronal functions, with both strictly required for circadian timekeeping in the SCN.

### Inhibition of Glutamate Uptake De-synchronizes the SCN Circuit

**Figure 2:**
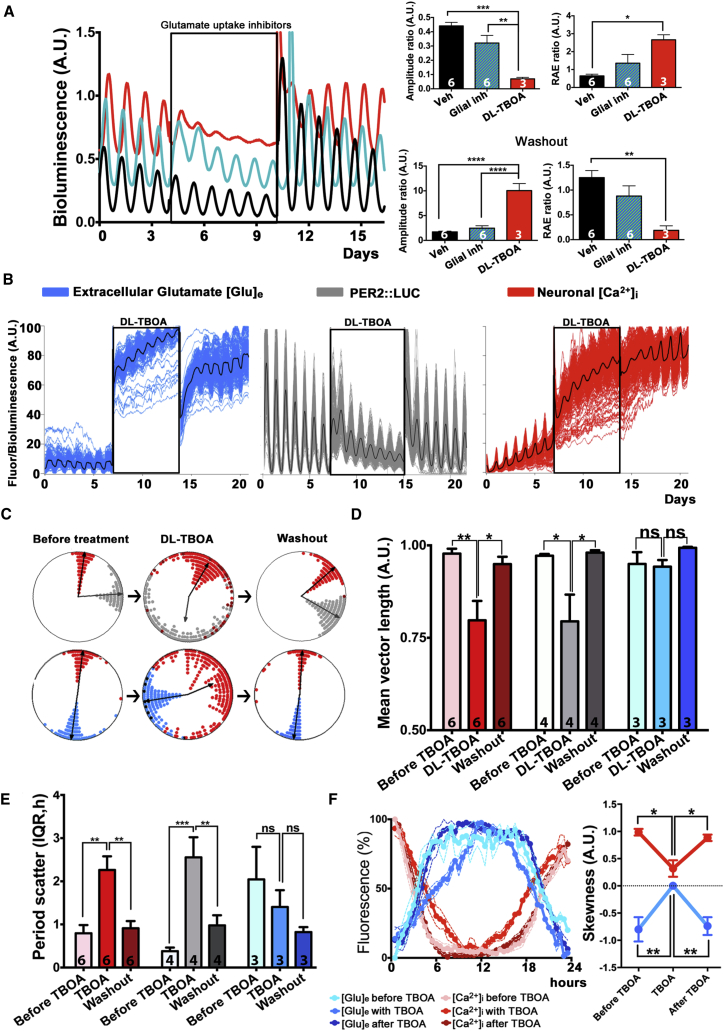
Glutamatergic Regulation of Circadian Synchrony of SCN Neurons (A) Representative PMT PER2::LUC traces and bar graphs of amplitude and RAE ratio of SCN slices treated with a cocktail of inhibitors of glial glutamate transporters (EAAT1 and EAAT2) (20 μM UCPH-101 + 10 μM WAY-213613), or with DL-TBOA (200 μM), which also inhibits the neuronal EAAT3. (B) Representative multi-channel imaging traces of Syn-RCaMP1h, PER2::LUC, and Syn-iGluSnFR cellular oscillations plus mean traces (black lines) showing effects of DL-TBOA treatment on these reporters. (C and D) Representative Rayleigh plots (C) and bar graphs of mean vector length (D) showing Syn-RCaMP1h, PER2::LUC, and iGluSnFR oscillators before DL-TBOA treatment, in the presence of the drug and after washout (N_osc/SCN_ > 100, n = 3). (E) Period scatter of RCaMP1h, PER2::LUC, and iGluSnFR cellular reports in SCN treated with DL-TBOA. (F) Circadian waveforms of [Glu]_e_ and [Ca^2+^]_i_, and corresponding estimates of skewness for SCN treated with DL-TBOA. Bar graphs are mean ± SEM; n experimental replicates depicted on bars. Statistical tests are as follows: two-way repeated-measures ANOVA, Bonferroni corrected. ^∗^p < 0.05; ^∗∗^p < 0.01; ^∗∗∗^p < 0.001; ^∗∗∗∗^p < 0.0001. See also Figure S5.

To test for a specific role of the astrocytically released [Glu]_e_ in mediating SCN circuit-level timekeeping, we inhibited excitatory amino acid transporters (EAAT) to block glutamate uptake (Jensen et al., 2015) and thereby dysregulate [Glu]_e_ levels. Pharmacological inhibition of the main glial isoforms of the transporters (EAAT1 and EAAT2), alone or in combination, did not significantly alter PER2::LUC rhythms (Figures 2A, S5A, and S5B). In contrast, treatment with DL-TBOA, which blocks neuronal (EAAT3) isoforms as well as the glial transporters, dramatically reduced the amplitude and robustness of PER2::LUC oscillations (Figures 2A, S5C, and S5D). Interestingly, EAAT3 mRNA expression is circadian in the SCN (Cagampang et al., 1996) and the transporter is strongly expressed by SCN GABAergic neurons (Figure S5E). We then followed circadian cycles of gene expression, neuronal [Ca^2+^]_i_, and [Glu]_e_ in SCN treated with DL-TBOA. As anticipated, DL-TBOA immediately increased [Glu]_e_, consistent with blockade of EAAT-mediated glutamate uptake (Figure 2B). Following this increase, individual neuronal rhythms of PER2::LUC and [Ca^2+^]_i_ were strongly de-synchronized and their periods scattered. Neuronal timekeeping, therefore, was compromised by EAAT blockade. Surprisingly, DL-TBOA did not significantly alter phase synchrony and period distribution of cellular reports of [Glu]_e_ (Figures 2C–2E). [Glu]_e_ circadian variations were, however, reshaped to more symmetrical waveforms, as quantified by reduced skewness in single cells and their aggregate signal. Neuronal [Ca^2+^]_i_ and the PER2::LUC waveforms were changed likewise, mirroring [Glu]_e_ (Figures 2F and S5F). We interpreted this as evidence of continuing circadian oscillation of glutamate release by astrocytes: unveiled by blockade of glutamate uptake but not de-synchronized by it. These results suggest that [Glu]_e_ oscillations are generated by concerted rhythms of release and uptake, and that blocking glutamate uptake impairs the fine-tuning of the [Glu]_e_/[Ca^2+^]_i_ relationship, thus reducing the sharpness of the rhythms of neuronal [Ca^2+^]_i_ across the SCN. Consequently, SCN cellular oscillators progressively de-synchronize, until the drug is removed and the [Glu]_e_/[Ca^2+^]_i_ alignment restored.

### NMDAR2C Mediates Glutamatergic Gliotransmission in the SCN

**Figure 3:**
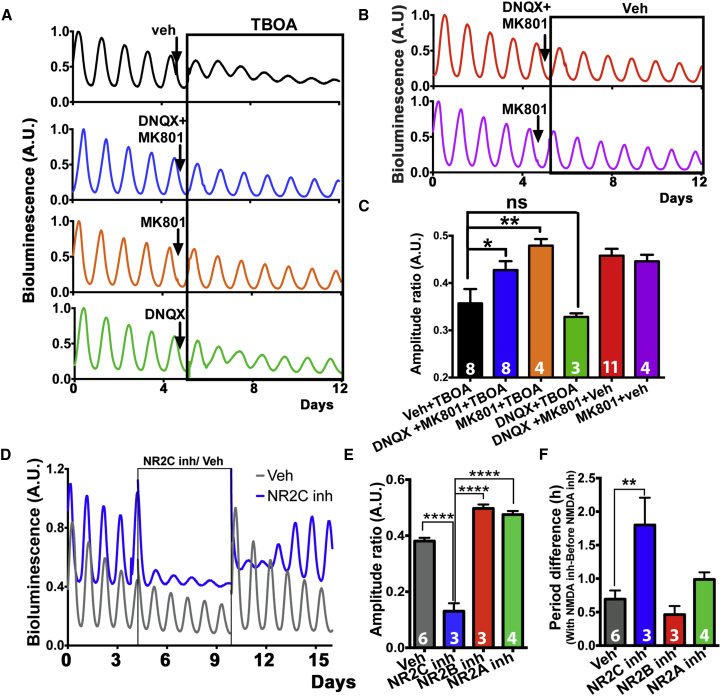
NMDAR Assemblies Containing NR2C Mediate Effects of Extracellular Glutamate on Circadian Rhythmicity in SCN Slices (A and B) Representative PMT traces from SCN slices expressing PER2::LUC pre-treated with MK-801 (12.5 μM) and DNQX (12.5 μM), alone or in combination, before DL-TBOA (A) or vehicle addition (B). (C) Bar graphs showing effects of pre-treatment with MK-801, alone or in combination with DNQX in slices treated with DL-TBOA or vehicle, respectively. (D) Representative PMT traces of PER2::LUC SCN slices treated with the NR2C antagonist DQP-1105 (50 μM) or vehicle. (E and F) Bar graphs showing (E) amplitude ratio and (F) period difference in SCN slices treated with DQP-1105, when compared to slices treated with vehicle and NR2A or NR2B antagonists (1 μM TCN-201 and 1 μM Ro-25-6981, respectively). All bar graphs mean ± SEM; n experimental replicates depicted on bars. Statistical test is as follows: (A–F) two-way repeated-measures ANOVA, Bonferroni corrected; n experimental replicates depicted on bars. ^∗^p < 0.05; ^∗∗^p < 0.01; ^∗∗∗∗^p < 0.0001. See also Figure S6.

We then investigated the neuronal receptors responsible for translating the dysregulation caused by compromised [Glu]_e_. Given the persistent de-synchronization of the SCN neuronal circuit in the presence of TBOA, we focused on NMDARs as ideal candidates in mediating long-term plastic effects of [Glu]_e_. Consistent with this view, pre-treating SCN slices with the NMDAR blocker MK-801 prevented the effects of DL-TBOA on PER2::LUC oscillations (Figures 3A and 3C), thus showing that activation of NMDARs is strictly necessary to mediate the de-synchronization of the SCN circuit caused by elevated [Glu]_e_ in the presence of DL-TBOA. In contrast, AMPA and Kainate glutamate receptors neither counteracted the damping of the PER2::LUC rhythms, nor enhanced MK-801 effects, as shown by treatment with DNQX alone or in combination with MK-801, respectively (Figures 3A and 3C). Thus, NMDARs are specifically responsible for mediating the dysregulatory effects elicited by the increase of [Glu]_e_. MK-801 on its own did not, however, damp PER2::LUC oscillations (Figure 3B), potentially suggesting that although higher [Glu]_e_ triggered by DL-TBOA may act via NMDAR, lower physiological [Glu]_e_ may not be sufficient to activate it. Alternatively, MK-801 has poor efficacy against NMDAR assemblages specifically containing NMDAR2C/D subunits (NR2C/D) in the mouse (Kutsuwada et al., 1992). NMDAR subunit composition varies widely across brain regions, and many areas of the brain express various combinations of the NMDAR1, 2A, 2B, 2C, and 2D subunits (Paoletti et al., 2013). Moreover, whereas NR2A and B are highly expressed in the hypothalamus, NR2C is specifically restricted to the dorsal SCN (Moriya et al., 2000, O’Hara et al., 1995, Watanabe et al., 1993) (Allen Brain Atlas Grin2c-RP_060502_01_A02) and NR2D is virtually absent. This pattern of NR2C expression resembles that of [Ca^2+^]_i_ localized in astrocytic microdomains (Figure 1C). Moreover, NR2C are less sensitive than NR2A and NR2B to voltage-mediated Mg^2+^ blockade (Clark and Kofuji, 2010, Mikkelsen et al., 1995, Paoletti et al., 2013) and so would remain sensitive to [Glu]_e_ during circadian night, when SCN neurons are hyperpolarized and astrocytically derived [Glu]_e_ is high (Figures 1A, 1D, and 1E). We therefore hypothesized that under physiological conditions, the MK-801-insensitive NR2C subunit could mediate astrocytic-neuronal glutamatergic signaling, whereas higher [Glu]_e_ levels, as elicited by DL-TBOA, would also activate MK-801-sensitive NR2A and NR2B. To test this, we treated SCN slices with the NR2C-selective antagonist DQP-1105 (Acker et al., 2011). This greatly reduced the overall amplitude and lengthened the period of the PER2::LUC oscillations, effects reversed on drug removal. In contrast, NR2A- and NR2B-selective antagonism neither reduced the amplitude, nor lengthened the period of PER2::LUC oscillations (Figures 3D–3F, S6A, and S6B). To confirm NR2C involvement, a structurally unrelated antagonist, QNZ-46 (Hansen and Traynelis, 2011), was also applied, and this replicated the effects of DQP-1105, thereby excluding putative off-target effects (Figures S6C and S6D).

### NR2C Expression Defines a Dorsal Microcircuit Required to Sustain Circadian Timekeeping in the SCN

**Figure 4:**
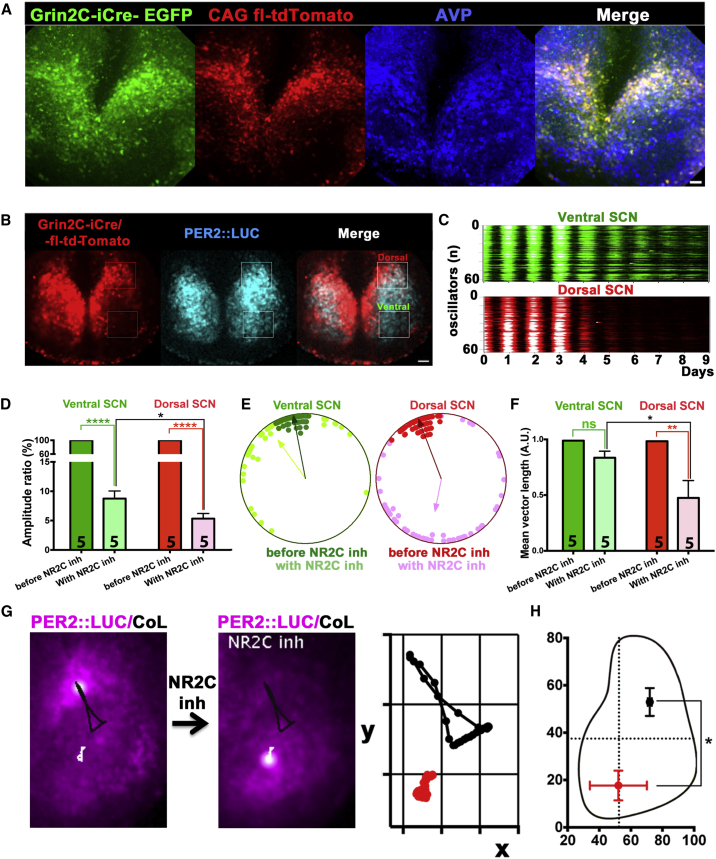
NR2C Inhibition Alters Spatiotemporal Wave of Clock Gene Expression by Selectively Impairing Synchronization of Dorsal NR2C^+^ SCN Neurons (A) Confocal micrographs of SCN slices from *Grin2C-iCre* mice transduced with AAV-CAG-flex-tdTomato and counterstained with AVP antiserum to highlight SCN cytoarchitecture. (B) Representative frame from time-lapse recording of PER2::LUC SCN slices from *Grin2C-iCre* mice expressing flex-tdTomato to label NR2C^+^ neurons. Boxed areas show dorsal and ventral SCN, as defined by tdTomato signal. (C and D) Raster plots (C) and bar graphs (D) of amplitude ratio of PER2::LUC oscillations in ventral and dorsal regions of SCN slices treated with DQP-1105. (E and F) Representative Rayleigh plots (E) and bar graphs (F) of mean vector length of ventral and dorsal PER2::LUC oscillators, as defined by Grin2C-tdTomato expression, before DQP-1105 treatment and in the presence of the drug. (G and H) (G) Photomicrographs and Poincaré plots of spatiotemporal waves of PER2::LUC quantified by CoL before (black trajectory) and during (white trajectory; red on Poincaré plot) DQP-1105 treatment, within a single SCN and across several replicates (H). SCN outline is for presentation purposes. All bar graphs are mean ± SEM; n experimental replicates depicted on bars. Statistical tests are as follows: (D and F) two-way repeated-measures ANOVA, Bonferroni corrected; (G) paired two-tailed t test, n = 3. ^∗^p < 0.05; ^∗∗^p < 0.01; ^∗∗∗∗^p < 0.0001. Scale bars, 50 μm. See also Figure S7 and Movie S2.

The circadian [Ca^2+^]_i_ signal localized in astrocytic microdomains in SCN slices showed a dorsal localization (Figure 1C), similar to NR2C expression patterns (Watanabe et al., 1993), thus suggesting that astrocyte-neuronal interactions may define a specific subcircuit in the dorsal SCN. We therefore tested if NR2C inhibition would selectively compromise the circuit-dependent spatiotemporal wave of clock gene expression in the dorsal SCN, as quantified by center of luminescence (CoL) analysis (Brancaccio et al., 2013). We first confirmed dorsal expression of NR2C by using SCN slices from *Grin2C-CreERT2-F2A-EGFP* (*Grin2C-iCre*) knockin Cre-driver mice, which express the inducible Cre recombinase together with an independent EGFP tag under the control of the NR2C promoter *Grin2C*. Heterozygous *Grin2C-iCre* slices were transduced by AAVs expressing a flexed tdTomato marker and Cre was induced with 4-OH-tamoxifen to confirm dorsal expression of NR2C and that the SCN cyto-architecture was preserved (Figure 4A). The efficiency of recombination of the CAG-flex-tdTomato AAV vector in Grin2C-CreERT2-F2A-EGFP^+^ cells was 99.5% (N_Grin2C-CreERT2-F2A-EGFP_^+^ = 959, n = 3), whereas no CAG-flex-tdTomato was expressed in the absence of EGFP signal. We then confirmed the neuronal nature of Grin2C-tdTomato^+^ cells and characterized the electrophysiological properties of this newly identified SCN neuronal subpopulation (Figure S7). Finally, to test for the involvement of NR2C neurons in defining the spatiotemporal wave, we treated *Grin2C-iCre* slices with DQP-1105 and compared the effects of NR2C antagonism in the Grin2C-positive and Grin2C-negative SCN regions (as defined by tdTomato expression). We found that although the amplitude of the cellular reports of PER2::LUC was overall reduced, this effect was significantly more prominent in the NR2C-positive dorsal SCN than in the NR2C-impoverished ventral region (Figures 4A–4D). Moreover, phase dispersal of PER2::LUC oscillators showed significant de-synchronization in the dorsal SCN in the presence of the NR2C antagonist, but not in the ventral SCN (N_osc/dSCN_ ≥ 40; N_osc/vSCN_ ≥ 40; n = 5) (Figures 4E and 4F). We reasoned that the impaired synchronization of the dorsal SCN triggered by interference with the [Glu]_e_/NR2C axis may specifically alter the spatiotemporal TTFL wave by selectively disorganizing the dorsal SCN. CoL analysis on PER2::LUC-expressing slices treated with DQP-1105 confirmed that the spatiotemporal wave of PER2::LUC was, in fact, dramatically re-programmed, showing a distinct ventralization (Figures 4G and 4H; Movie S2), consistent with the selective depression of a dorsal circuit module incorporating the astrocytic [Glu]_e_-NR2C intercellular axis.

### A Model for the Astrocytic-Neuronal Intercellular Axis Sustaining Circadian Timekeeping in the Dorsal SCN Microcircuit

**Figure 5:**
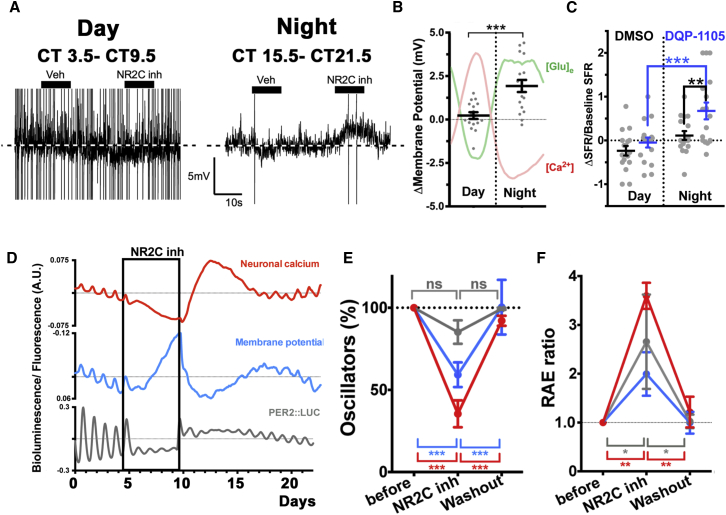
NR2C Inhibition Depolarizes SCN Neurons and Impairs Coherence of Circadian Oscillations of Membrane Potential, [Ca^2+^]_i_, and Clock Gene Expression (A) Representative electrophysiological traces showing membrane potential recorded from dorsal SCN neurons during the circadian day (CT3.5–CT9.5) or night (CT15.5–CT21.5). Black bars indicate serial vehicle and DQP-1105 treatments. Traces rescaled to highlight the extent of DQP-1105-induced depolarization. (B) Scatterplots of change in membrane potential during focal DQP-1105 application relative to DMSO (mean ± SEM; CT3.5–CT9.5, n = 17; CT15.5–CT21.5, n = 18; each time point sampled from four SCNs; unpaired two-tailed t test). Projected circadian oscillations of neuronal [Ca^2+^]_i_ and [Glu]_e_ are shown for presentation purposes. (C) Scatterplots of change in spontaneous firing rates (SFRs), during either DQP-1105 or DMSO treatment, normalized to baseline SFR, recorded immediately before treatment (two-way repeated-measures ANOVA, with Bonferroni correction; n = 17). (D) Representative aggregate traces showing changes of simultaneously recorded neuronal [Ca^2+^]_i_, membrane potential, and PER2::LUC, induced by DQP-1105 and after washout. (E and F) Bar graphs showing severe reduction in the number (E) and quality (high RAE) (F) of oscillations of neuronal [Ca^2+^]_i_, membrane potential, and PER2::LUC at single-cell level (mean ± SEM, N_osc/SCN_ > 60, n = 3; two-way repeated-measures ANOVA, Bonferroni corrected; ^∗∗∗^p < 0.001).

**Figure 6:**
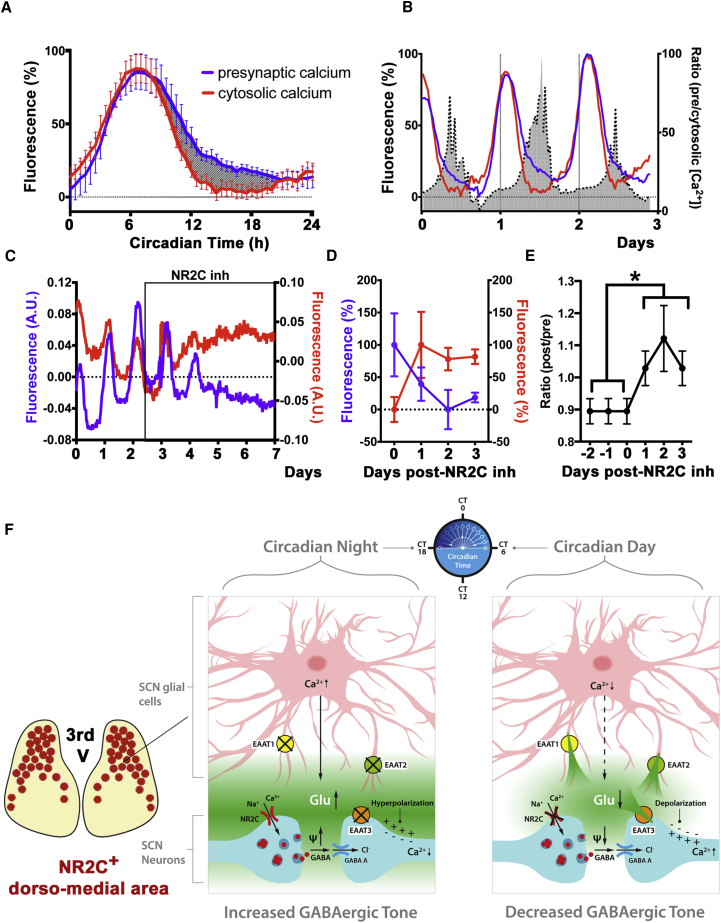
NR2C Antagonism Decouples Pre-synaptic and Cytosolic [Ca^2+^]_i_ in SCN Neurons (A and B) (A) Circadian profiles (mean ± SEM, n = 5) of SCN slices co-transduced with SyF::GCaMP3 and Syn-RCaMP1h AAVs to detect pre-synaptic or cytosolic [Ca^2+^]_i_, respectively. Gray area shows higher nighttime fluorescence of pre-synaptic [Ca^2+^]_i_ in comparison to cytosolic [Ca^2+^]_i_ and circadian variation of pre-synaptic/cytosolic neuronal [Ca^2+^]_i_ ratios (B). (C) Representative traces of SyF::GCaMP3 and Syn-RCaMP1h fluorescence in SCN slices before and during DQP-1105 treatment. (D) Mean ± SEM of baseline SyF::GCaMP3 and Syn-RCaMP1h fluorescence in the presence of DQP-1105. (E) Mean ± SEM ratios of cytosolic/pre-synaptic baseline fluorescence before DQP-1105 and in the presence of the drug (paired two-tailed t test; ^∗^p < 0.05, n = 5). (F) Cartoon depicting the proposed model for the astrocytic-neuronal intercellular axis in the dorsal SCN.

To test if [Glu]_e_ may be directly involved in mediating an inhibitory astrocytic role in the dorsal SCN through NR2C inhibition, and, if so, to elucidate the mechanisms of such inhibition, we performed electrophysiological recording in SCN slices treated with NR2C antagonists. Remarkably, NR2C antagonism depolarized dorsal SCN neurons and increased their spontaneous firing rates specifically during circadian night when [Glu]_e_ is high, but was ineffective during the day when [Glu]_e_ is low (Figures 5A–5C). Long-term imaging of DQP-1105-treated slices confirmed that membrane potential, simultaneously recorded alongside PER2::LUC, was increased over several days in the presence of DQP-1105, consistent with the acute electrophysiological responses. Furthermore, DQP-1105 also abolished circadian oscillations of [Ca^2+^]_i_ in the majority of SCN neurons (64.5% ± 8% arrhythmic cells; N_osc/SCN_ > 200; n = 3; p < 0.001) and decreased the robustness of membrane potential, [Ca^2+^]_i_, and PER2::LUC residual oscillations (Figures 5D–5F). This demonstrated that NR2C-mediated glutamatergic gliotransmission inhibits neuronal activity during circadian night and that this is essential to sustain circadian rhythmicity in the dorsal SCN. To reconcile the well-known excitatory role of NMDAR activation with the apparently paradoxical stimulatory effects of NR2C antagonism on SCN neurons, we hypothesized that NR2C could be located pre-synaptically in the dorsal SCN and that its activation would facilitate release of the inhibitory neurotransmitter GABA during the circadian night, a time when neuronal [Ca^2+^]_i_ is low, thereby inhibiting postsynaptic neurons. To test this, we monitored pre-synaptic [Ca^2+^]_i_ by transducing SCN slices with AAVs encoding Synaptophysin::GCaMP3 (Syf:GCaMP3) reporter, in which GCaMP3 is fused to Synaptophysin to localize it to synaptic vesicles (Figure 6). Although pre-synaptic [Ca^2+^]_i_ was rhythmic and shared the same overall phase of the cytosolic neuronal [Ca^2+^]_i_, waveform analysis revealed sustained levels of nighttime pre-synaptic [Ca^2+^]_i_, which were absent in the cytosolic neuronal [Ca^2+^]_i_. This created a circadian variation in the pre-synaptic/cytosolic [Ca^2+^]_i_ ratio in SCN neurons (Figures 6A and 6B), data that are consistent with the idea that nighttime glutamate released by astrocytes would sustain higher [Ca^2+^]_i_ specifically in the pre-synaptic terminals, due to NR2C-dependent activation. Furthermore, we reasoned that if NR2C is effectively localized pre-synaptically and facilitates GABA release, then treating SCN slices with DQP-1105 should decouple pre-synaptic [Ca^2+^]_i_ from postsynaptic [Ca^2+^]_i_, by decreasing inhibitory GABA release and thus simultaneously increasing postsynaptic cytosolic [Ca^2+^]_i_. Indeed, NR2C antagonism elicited an immediate and sustained decrease in pre-synaptic SyF::GCaMP3 signal, whereas it increased cytosolic [Ca^2+^]_i_, co-detected by Syn-RCaMP1 (Figures 6C–6E). This demonstrates a pre-synaptic action of NR2C blockade and suggests how such blockade would cause a paradoxically excitatory effect in neurons of the dorsal SCN: during nighttime, extracellular glutamate from astrocytes enhances circuit-wide inhibitory tone by activating pre-synaptic NMDAR2C in GABAergic neurons (Figure 6F).

### Temporal Misalignment of Astrocytes and Neurons Reveals a Role for SCN Astrocytes in Encoding Spatiotemporal Circadian Information

**Figure 7:**
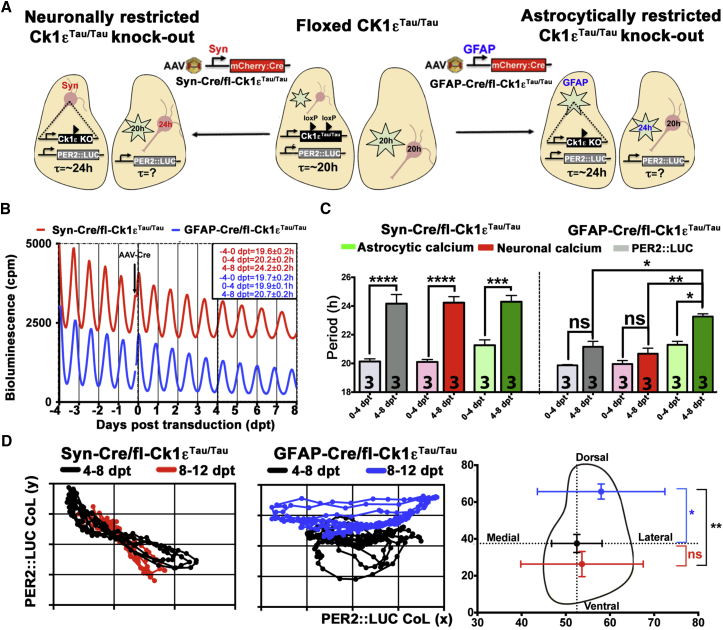
SCN Astrocytes Control Spatiotemporal Wave of Circadian Gene Expression in Juvenile Slices (A) Schematic of strategy, with floxed Ck1ε^Tau/Tau^ PER2::LUC SCN transduced with AAVs encoding Cre recombinase driven by Syn or GFAP promoters to assess the effects of neuronal and astrocytic Ck1ε^Tau/Tau^ knockout on circadian rhythms. (B) Representative PMT traces of PER2::LUC SCN from neuronal- or astrocyte-restricted Ck1ε^Tau/Tau^ knockout (mean ± SEM, p < 0.0001, n = 4). (C) Bar graphs showing period of PER2::LUC, neuronal, and astrocytic [Ca^2+^]_i_ circadian oscillations co-detected in neuronal- or astrocyte-restricted Ck1ε^Tau/Tau^ knockout, respectively. (D) Poincaré plots revealing CoL dorsalization in astrocytic-restricted Ck1ε^Tau/Tau^ knockout, within a single SCN (left) and across different SCNs (right, n = 3; ANOVA). SCN outlined for presentation purposes. Bar graphs are mean ± SEM; n experimental replicates as depicted on bars. Statistical test is as follows: two-way repeated-measures ANOVA, Bonferroni corrected; ^∗^p < 0.05; ^∗∗^p < 0.01; ^∗∗∗^p < 0.001; ^∗∗∗∗^p < 0.0001. dpt, days post-transduction. See also Movie S3.

To further explore the contribution of astrocytes to circuit-level timekeeping, we specifically manipulated astrocytic or neuronal cell-autonomous TTFLs. Floxed *Ck1ε*^*Tau/Tau*^ mice show ∼20 hr TTFL rhythms that can be reverted to ∼24 hr by Cre-mediated excision of the targeted allele. By directing Cre expression to neurons or astrocytes in SCN slices (by using the Syn or GFAP promoters, respectively) (Figure S3), we aimed to create temporally chimeric SCN in which neurons had 24 hr (*Ck1ε*^*Tau*^-deleted) and astrocytes had 20 hr TTFLs, or vice versa (Figure 7A). This temporal misalignment was designed to reveal any reciprocal interactions between neurons and astrocytes relevant for circadian pacemaking. After a lag of about 4 days from AAV transduction, due to the cycle of AAV activity (Figure S3C) (as previously observed for similar period manipulations) (Edwards et al., 2016), neuronally restricted *Ck1ε*^*Tau*^ deletion lengthened the period of neuronal PER2::LUC oscillations to 24 hr, whereas GFAP-restricted deletion did not, thus confirming the primacy in the slice of the neuronal TTFL over astrocyte-derived signals in setting the ensemble circadian period (Figure 7B). However, simultaneous recording of neuronal and astrocytic [Ca^2+^]_i_ rhythms revealed a more complex picture of astrocytic-neuronal interdependence. Whereas neuronal deletion of *Ck1ε*^*Tau*^ lengthened the period of both astrocytic and neuronal [Ca^2+^]_i_ oscillations, astrocytically restricted *Ck1ε*^*Tau*^ deletion selectively lengthened the period of astrocytic [Ca^2+^]_i_, without significantly affecting neuronal [Ca^2+^]_i_ (Figure 7C). Astrocyte-restricted *Ck1ε*^*Tau*^ deletion thus created a temporally misaligned SCN: a circuit in which the neuronal and astrocytic populations co-existed with distinct cell-intrinsic periods. Given our previous observation of re-programming of the spatiotemporal wave of gene expression by NR2C antagonists (Figure 4) and the dorsal location of the [Glu]_e_/NR2C axis module (Figure 6F), we asked whether this area would selectively respond to changes in the altered periodic input arising from the astrocytic clockwork in chimeric SCN. Indeed, whereas neuronal *Tau* deletion did not affect the ensemble PER2::LUC spatiotemporal wave, astrocytically restricted *Ck1ε*^*Tau*^ knockout dramatically dorsalized it (Figure 7D; Movie S3). These results are consistent with the idea that astrocytic control of the SCN circuit is mediated by a dorsal neuronal subpopulation specifically capable of reading the new periodic input coming from the SCN astrocytes. This selective response creates a non-random dynamic reshaping of the intrinsic phase relationship among PER::LUC oscillators along the ventro-dorsal axis, detected by the CoL analysis. In contrast, neuronal periodic reprogramming is untargeted to any specific SCN subpopulations, and therefore, although the ensemble period is dramatically changed, the underlying internal network properties are unaffected, as their reciprocal phase relationships co-vary in time.

### SCN Astrocytes Control the Intrinsic Periodicity of Circadian Behavioral Rhythms in Adult Mice

**Figure 8:**
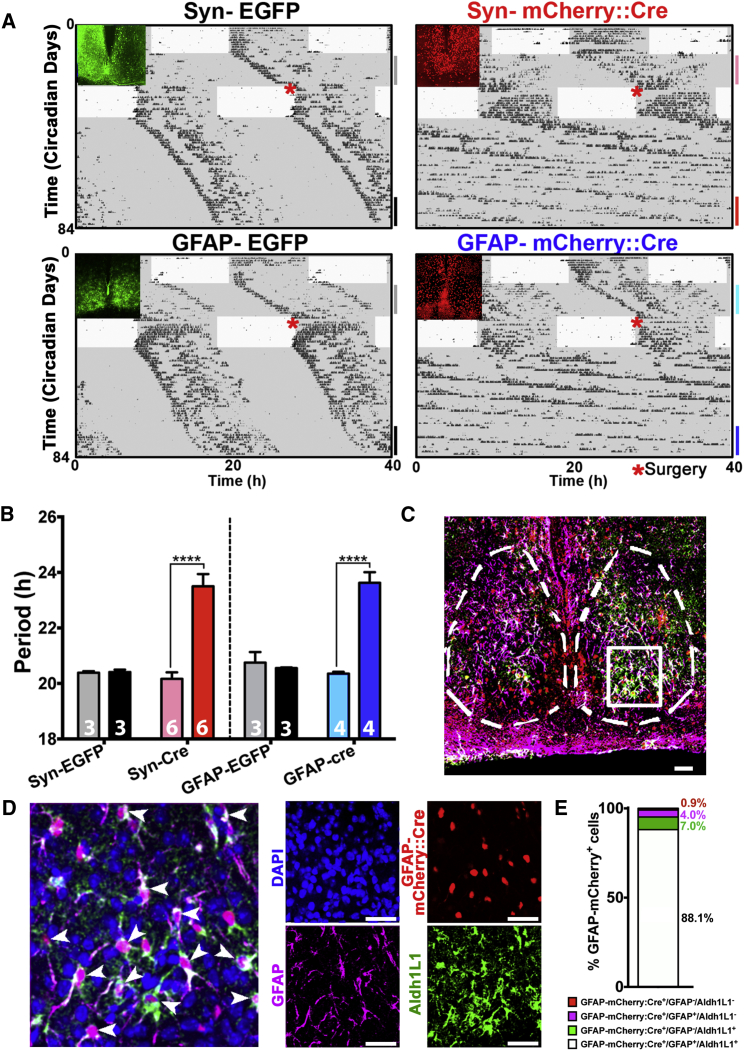
SCN Astrocytes Control Circadian Patterns of Locomotor Activity in Adult Mice (A) Representative double-plotted actograms of wheel-running behavior in Ck1ε^Tau/Tau^ mice in DD (gray area), following stereotaxic SCN targeting by AAVs expressing Syn- or GFAP-restricted Cre; absent in EGFP-expressing controls. Note period lengthening following surgery in both Syn- and GFAP-Cre mice. Insets show effective targeting of SCN corresponding to plotted actograms and distinct morphologies of Syn/GFAP-EGFP targeted SCN cells. (B) Bar graphs showing period in DD before and after stereotaxic surgery in Syn- and GFAP-Cre targeted mice and respective EGFP controls. Windows of period detection are color-coded as reported on the actograms. Bar graphs are mean ± SEM; n experimental replicates depicted on bars. (C) Confocal micrographs showing counterstaining of GFAP-mCherry::Cre with the astrocytic markers GFAP and Aldh1L1. (D) Inset: merge showing co-localization of mCherry^+^ with GFAP and Aldh1L1 (arrows) in SCN targeted area as highlighted in (C). Fluorescent signals also presented as single channels. (E) Co-localization rates of GFAP-mCherry::Cre^+^ cells with the astrocytic markers GFAP and Aldh1L1 (N_GFAP-mCherry::Cre_^+^ = 328; n = 3). Statistical test is as follows: two-way repeated-measures ANOVA, Bonferroni corrected; ^∗∗∗∗^p < 0.0001. Scale bars, 50 μm. See also Figure S8.

Surprisingly, although GFAP-restricted deletion of *Ck1ε*^*Tau*^ lengthened astrocytic [Ca^2+^]_i_ period and altered the spatiotemporal wave in the dorsal SCN, it did not significantly affect the overall period of PER2::LUC oscillations (Figure 7B). This, however, could reflect the nature of the preparation (isolated juvenile SCN slices), rather than the full extent of the SCN astrocytic function. In fact, it has been shown in other brain areas that during the early postnatal period, astrocytes are more involved in establishing neural networks, whereas as they mature they acquire more complex functional roles by regulating metabolic and synaptic activation (Molofsky and Deneen, 2015). It is therefore possible that although SCN astrocytes can already affect the circuit behavior in the early postnatal period (Figure 7D; Movie S3), pacemaking function could require their full functional maturation. To test if SCN astrocytes can control intrinsic circadian periodicity in the adult mouse and in more physiological conditions, we repeated the astrocytic-neuronal temporal misalignment experiments in vivo by stereotaxically targeting SCN of *Ck1ε*^*Tau/Tau*^ mice with AAVs expressing mCherry::Cre (or control EGFP) driven by Syn or GFAP promoters, respectively, and recorded circadian patterns of wheel-running locomotor activity in constant darkness (DD), before and after surgery (Figures 8A, 8B, and S8). All mice showed an ∼20 hr period in DD before surgery, and as expected, mice whose SCN was efficiently targeted (N_Syn-mCherry::Cre_/N_DAPI_ = 22% ± 0.3%; mean ± SEM, N_DAPI_ = 11,921; n = 3) by neuronally restricted Cre recombinase (as confirmed post mortem by mCherry::Cre expression in SCN sections) showed a dramatic period lengthening (∼4 hr) (Figures 8A, 8B, S8A, and S8E). Surprisingly, an equivalent lengthening of behavioral period was also seen in mice subject to astrocytically restricted Cre recombinase (N_GFAP-mCherry::Cre_/N_DAPI_ = 10.7% ± 0.5%; mean ± SEM, N_DAPI_ = 9,191; n = 3). The astrocytic nature of the GFAP-Cre::mCherry-expressing cells was confirmed in these mice by co-localization of GFAP-Cre::mCherry with the astrocytic markers GFAP and Aldh1L1 (Figures 8C–8E). Most GFAP-Cre::mCherry^+^ cells (∼81%) were also co-expressing GFAP, whereas no significant co-localization with GFAP could be observed in Syn-Cre::mCherry^+^ SCN (<1%) (Figures S8B and S8C). A minority of the GFAP-Cre::mCherry^+^ (∼19%) did not co-express detectable GFAP; however, we reasoned that this estimation may be affected by the spatial (Figure S8C) and circadian temporal complexity of GFAP staining in the SCN (Lavialle and Servière, 1993). Therefore, we also counterstained the GFAP-Cre::mCherry transduced SCN with a second astrocytic marker (Aldh1L1). In these experiments, not only did we find that ∼88% of the mCherry^+^ cells expressed both GFAP and Aldh1L1, but also that >99% of the mCherry^+^ cells expressed at least one astrocytic marker, thus confirming the high specificity of our manipulations (Figures 8C–8E). Accordingly, SCN targeted with the EGFP tag expressed by Syn or GFAP promoters in control animals showed distinct neuronal and astrocytic morphologies, respectively, as expected given non-overlapping expression from these promoters in SCN slices (Figures S8D and S3D), and the high specificity of the co-immunostaining with GFAP and Aldh1L1. Remarkably, these data demonstrate that not only SCN neurons, but also SCN astrocytes, are able to encode circadian information and control overt circadian behavior, showing the intrinsically dual nature of the master clock circuit in mammals.

## Discussion

Long-term imaging of [Ca^2+^]_i_ in SCN slices revealed complementary anti-phasic circadian cycles of neuronal and astrocytic activation, with [Ca^2+^]_i_ levels peaking during the circadian day and night, respectively. A general marker of metabolic activation in both astrocytes and neurons, [Ca^2+^]_i_ is commonly linked to release of glio- and neurotransmitters (Henneberger et al., 2010, Jourdain et al., 2007). We therefore hypothesized that during the circadian night, SCN astrocytes inhibit SCN neurons by releasing gliotransmitters into the extracellular space. The identification of self-sustained circadian oscillations of [Glu]_e_, anti-phasic to neuronal [Ca^2+^]_i_ and membrane potential, and so synchronous with astrocytic [Ca^2+^]_i_, suggested that [Glu]_e_ may provide this inhibitory astrocytic-neuronal coupling signal. The astrocytic origin of [Glu]_e_ was confirmed by pharmacological inhibition of astrocytic catabolism of glutamate by MSO, as well as by selective ablation of SCN neurons or astrocytes by genetically activated caspase 3, respectively increasing or decreasing [Glu]_e_. Pharmacological inhibition of glutamate uptake severely dysregulated neuronal circadian oscillations, an effect that was prevented by NMDAR blockade. In particular, selectively inhibiting NR2C damped circadian oscillations in the SCN and lengthened their period, highlighting a role for NR2C in sensing [Glu]_e_ oscillations. Selective expression of NR2C in the dorsal SCN suggests that this astrocytic [Glu]_e_/neuronal NR2C axis may confer region-specific properties to the SCN circuit. To test this, we genetically de-coupled the intrinsic periods of TTFLs of SCN astrocytes and neurons via astrocyte-specific deletion of *Ck1ε*^*Tau/Tau*^, or we pharmacologically inhibited NR2C. These manipulations elicited opposite and complementary displacements of the spatiotemporal wave of TTFL activation that could be attributed to the selective re-programming of the dorsal SCN. To demonstrate that NR2C directly mediates glutamatergic inhibition onto SCN neurons, we tested the effects of NR2C antagonism on membrane potential, neuronal [Ca^2+^]_i_, and clock gene expression. NR2C antagonism elicited nighttime depolarization of SCN dorsal neurons but was ineffective during the circadian day, thus confirming an overall nocturnal inhibitory role of NMDAR2C on dorsal SCN neurons. Prolonged NR2C antagonism not only increased membrane potential, but also disrupted circadian neuronal rhythms of membrane potential, [Ca^2+^]_i_, and clock gene expression. Thus, we propose a new working model of astrocytic-neuronal circadian pacemaking in the SCN. During the circadian night, SCN astrocytes are metabolically active (high [Ca^2+^]_i_) and release high levels of glutamate into the extracellular space, which in turn activates pre-synaptic NR2C-expressing neurons in the dorsal SCN, thereby increasing GABAergic inhibitory tone across the circuit (Kang et al., 1998). In contrast, during the circadian day [Glu]_e_ is reduced by diminished glial release and increased EAAT-mediated glutamate uptake and consequently GABAergic tone is reduced, thereby de-repressing spontaneous membrane potential, neuronal [Ca^2+^]_i_, and facilitating electrical firing. Consistent with this idea, although NMDAR2C antagonism increased cytosolic [Ca^2+^]_i_ in SCN neurons, it decreased co-detected pre-synaptic [Ca^2+^]_i_.

The dorsal SCN has been proposed as a privileged site to mediate timekeeping functions in mammals (Mieda et al., 2016) in constant conditions, as opposed to the ventral SCN, which is involved instead with processing of glutamatergic light inputs coming from the retina. It is therefore particularly interesting that this functional organization would be mirrored by the spatial distribution of the NR2A and B (ventral) and the NR2C subunits (dorsal). This suggests that whereas NR2A and B mediate responses to glutamatergic retinal synaptic input, NR2C may be specifically involved in regulating intrinsic SCN timekeeping by sensing local oscillations of astrocytically released glutamate. Indeed, manipulating the TTFL in SCN astrocytes (as well as neurons) in vivo lengthened the intrinsic period of the mouse in constant darkness, in the absence of glutamatergic retinal input. This shows that SCN astrocytes and neurons should be considered together as two arms of the mammalian pacemaker circuit, both equally able to impart timekeeping information to the rest of the body. Interestingly, the co-existence of a 20 hr neuronal clock and 24 hr astrocytic clock (and vice versa) does not lead to any linear computation (and spread) in the period of locomotor activity behavior, but rather to a discrete and coherent 4 hr period lengthening. This preferentiality may be due to the fact the TTFL clocks (and SCN circuit) are optimized to work at 24 hr and therefore would be advantaged in the chimeras over the 20 hr cells, regardless of which cell type has been targeted.

The newly identified astrocytic glutamate/neuronal NR2C axis may be a novel adaptation of the mammalian master clock, evolved to mediate a conserved nighttime inhibitory signal, required for internal synchronization of brain pacemakers. In *Drosophila*, dorsal pacemaker neurons are also inhibited by glutamate (Collins et al., 2014, Hamasaka et al., 2007, McCarthy et al., 2011), and it was recently predicted (Collins et al., 2014) that GABA and glutamate may be good candidates to mediate a synchronizing dusk signal in the SCN. Although our data support this view, they also point to a completely different and unexpected mechanism in mammals involving an intercellular astrocytic Glu/neuronal NR2C axis, rather than purely neuronal feedbacks impinging on inhibitory glutamatergic chloride channels (absent in mammals), or metabotropic glutamate receptors (mainly playing modulatory roles). When compared to invertebrates, which express only two subunits, mammals enjoy a more diverse repertoire of electrophysiological and cellular responses, employing no fewer than eleven distinct NMDAR subunits, differentially expressed across brain areas (Paoletti et al., 2013). Therefore, NR2C may have been recruited in the SCN to mediate an inhibitory glutamatergic signaling of astrocytic origin, thereby favoring nighttime release of an inhibitory neurotransmitter, GABA. This two-cell-type model for the regulation of GABAergic tone overcomes difficulties inherent to current models that emphasize a modulatory role exclusively exerted by neuronal GABAergic inhibition in the SCN. First, membrane depolarization of GABA cells should trigger synaptic release of GABA during daytime and therefore depress further neuronal activation across the circuit, thus perpetually locking the mammalian pacemaker in the off state. Second, inhibitory effects of synaptically released GABA are maximal during nighttime (Itri et al., 2004, Wagner et al., 1997), when neuronal membrane potential and firing rates are at their nadir and so synaptic GABA release should be minimal. Devolving control of GABA release to SCN astrocytes, via [Glu]_e_/NR2C axis, at once resolves both inconsistencies by making the timing of nocturnal GABA release independent of GABAergic neuronal activity. The temporal segregation of astrocytic and neuronal activities would therefore confer contrast enhancement, increasing the temporal precision in the dorsal SCN by making the circuit not solely dependent on prolonged periods of neuronal silencing and activation, but rather on a recursive rebound between activities of SCN astrocytic and neuronal elements. It will be important to establish if this intercellular loop is also present elsewhere in the brain, in particular in areas of strong NR2C expression (like olfactory bulb and cerebellum), also characterized by strong circadian rhythmicity.

Our findings speak to unresolved questions regarding the tailoring of astrocytic function in different brain areas to selectively facilitate circuit control of distinct behaviors (Khakh and Sofroniew, 2015). Although astrocytes have been variously implicated in the expression of sleep-wake states, current reports mainly focus on homeostatic control of sleep (Pelluru et al., 2016, Petit and Magistretti, 2016). Our data instead demonstrate a purely circadian role of SCN astrocytes, independent of light/dark cycles and/or sleep centers, as shown by changes of circadian periodicity elicited by SCN astrocytes in constant darkness. It is therefore likely that the particular astrocytic contingents of different brain areas may play distinct roles on the circadian and homeostatic control of sleep. It is, however, remarkable that extracellular glutamate secreted by astrocytes seems to play a similar role as a synchronizing agent across different timescales, as shown for cortical slow waves (Poskanzer and Yuste, 2016), and now for hypothalamic SCN circadian waves, suggesting a unifying timing property for it in oscillatory function.

## STAR★Methods

### Key Resources Table

**Table:** 

REAGENT or RESOURCE	SOURCE	IDENTIFIER
**Antibodies**		

Anti-GFAP antibody, Chicken polyclonal to GFAP	Abcam	ab4674; RRID: AB_304558
Anti-Aldh1L1 antibody, Rabbit polyclonal to Aldh1L1	Abcam	ab87117; RRID: AB_10712968
Anti-GAD65 + GAD67, Rabbit polyclonal to GAD65 + GAD67	Abcam	ab49832; RRID: AB_880149
Anti-EAAT3 (EAAC1), Goat polyclonal to EAAC1	Millipore	AB1520; RRID: AB_90732
Anti-Ng2, Rabbit polyclonal to Ng2	Abcam	ab83178; RRID: AB_10672215
Anti-Iba1, Rabbit polyclonal to Iba1	Wako Chemicals	019-19741; RRID: AB_839504
Anti-AVP, Rabbit polyclonal to AVP	Bachem	T4562.0400; RRID: AB_518671
Alexa Fluor 647 Goat Anti-Chicken antibody	Thermo Fisher Scientific	A-21449; RRID: AB_2535866
Alexa Fluor 488 Goat Anti-Rabbit antibody	Thermo Fisher Scientific	A-11008; RRID:AB_143165
Alexa Fluor 488 Goat Anti-Chicken antibody	Thermo Fisher Scientific	A-11039; RRID: AB_142924
Alexa Fluor 647 Donkey Anti-Goat antibody	Thermo Fisher Scientific	A-21447; RRID: AB_141844
Alexa Fluor 488 Chicken Anti-Rabbit antibody	Thermo Fisher Scientific	A-21441; RRID: AB_141735
Alexa Fluor 647 Goat Anti-Rabbit antibody	Thermo Fisher Scientific	A-21245; RRID: AB_141775

**Chemicals, Peptides, and Recombinant Proteins**		

DL-TBOA (DL-*threo*-β-Benzyloxyaspartic acid)	Tocris Bioscience	Cat. No. 1223; CAS No 205309-81-5
UCPH-101 (2-Amino-5,6,7,8-tetrahydro-4-(4-methoxyphenyl)-7-(naphthalen-1-yl)-5-oxo-4*H*-chromene-3-carbonitrile)	Tocris Bioscience	Cat. No. 3490; CAS No 1118460-77-7
WAY 213613 *N*-[4-(2-Bromo-4,5-difluorophenoxy)phenyl]-L-asparagine	Tocris Bioscience	Cat. No. 2652; CAS No: 868359-05-1
DQP-1105 5-(4-Bromophenyl)-3-(1,2-dihydro-6-methyl-2-oxo-4-phenyl-3-quinolinyl)-4,5-dihydro-γ-oxo-1*H*-pyrazole-1-butanoic acid	Tocris Bioscience	Cat. No. 4491; CAS No: 380560-89-4
QNZ-46 4-[6-Methoxy-2-[(1*E*)-2-(3-nitrophenyl)ethenyl]-4-oxo-3(4*H*)quinazolinyl]benzoic acid	Tocris Bioscience	Cat. No. 4801; CAS No: 1237744-13-6
TCN-201 3-Chloro-4-fluoro-*N*-[4-[[2-(phenylcarbonyl)hydrazino]carbonyl]benzyl]benzenesulfonamide	Tocris Bioscience	Cat. No. 4154; CAS No: 852918-02-6
(+)-MK 801 maleate (5S,10R)-(+)-5-Methyl-10,11-dihydro-5H-dibenzo[a,d]cyclohepten-5,10-imine maleate	Tocris Bioscience	Cat. No. 0924; CAS No: 77086-22-7
MSO L-S-(3-Amino-3-carboxypropyl)-S-methylsulfoximine	Sigma-Aldrich	Cat. No. M5379; CAS No: 15985-39-4

**Experimental Models: Organisms/Strains**		

*Grin2c*^*tm1CreERT2_EGFP/Wtsi*^	Wellcome Trust Sanger Institute	MGI:5632501
Ck1e Tau (B6.129-Csnk1etm1Asil/J)	Jax Laboratories	RRID: IMSR_JAX:016183
PER2::LUC (B6.129S6-Per2tm1Jt/J)	Jax Laboratories	RRID: IMSR_JAX:006852

**Recombinant DNA**		

Syn.RCaMP1h.WPRE.SV40	Penn Vector Core	AV-1-PV3010
hSyn.GCaMP3.WPRE.SV40	Penn Vector Core	AV-1-PV1627
hSyn.iGluSnFr.WPRE.SV40	Penn Vector Core	AV-1-PV2723
GFAP.iGluSnFr.WPRE.SV40	Penn Vector Core	AV-1-PV2726
hSyn.ArcLightD.WPRE.SV40	Penn Vector Core	AV-1-36857P
GfaABC1D.PI.cyto-GCaMP3.SV40	Penn Vector Core	AV-5-44331
GfaABC1D.Lck-GCaMP6f.SV40	Penn Vector Core	AV-5-PV3107
AAV1.CMV.PI.SynGCaMP3.SV40	Penn Vector Core	AV-1-PV2368
CAG.Flex.tdTomato.WPRE.bGH	Penn Vector Core	AV-1-ALL864
hSyn.eGFP.WPRE.bGH	Penn Vector Core	AV-1-PV1696
GFAP.eGFP.WPRE.bGH	Penn Vector Core	AV-5-PV2407
EF1a.flex.EYFP.WPRE.hGH	Penn Vector Core	AV-1-27056
GFAP-hM3DGq:mCherry	UNC Vector Core	N/A
GFAP-mCherry:Cre	UNC Vector Core	N/A
Syn-mCherry:Cre	UNC Vector Core	N/A
GFAP-EGFP:Cre	UNC Vector Core	N/A
AAV-EF1a-flex-taCasp3-TEVp	UNC Vector Core	N/A

**Software and Algorithms**		

BioDare	(Moore et al., 2014)	https://www.biodare.ed.ac.uk
Clock Lab Analysis 6	ActiMetrics	http://actimetrics.com/products/clocklab/
Prism 6	GraphPad	http://www.graphpad.com/scientific-software/prism/
Igor Pro	WaveMetrics	https://www.wavemetrics.com/products/igorpro/igorpro.htm
SARFIA Semi-Automated Routines for Functional Image Analysis (Igor Plugin)	(Dorostkar et al., 2010)	http://www.igorexchange.com/node/1723
Axograph X	Axograph Scientific	http://www.axograph.com/
Neuromatic (Igor plugin)	Jason Rothman, UCL	http://neuromatic.thinkrandom.com
Oriana 4	Kovacs Computer Services	https://www.kovcomp.co.uk/oriana/

### Contact for Reagent and Resource Sharing

Further information and requests for reagents may be directed to and will be fulfilled by the Lead Contact, Michael H. Hastings (mha@mrc-lmb.cam.ac.uk).

### Experimental Model and Subject Details

#### Mice

All the experiments were performed on healthy mice, with normal immune status, housed in a specific pathogen free (SPF) unit (Ares Facility, Babraham Institute Campus, Cambridge, UK). Experimental subjects were not involved in any previous test or drug treatment. For ex vivo SCN slice experiments, both female and male pups were used. Pups were maintained in a 12:12 light-dark cycle together with their mothers before being sacrificed at P12-P14. Food and water was provided ad libitum. In vivo wheel-running behavior was recorded in individually caged (8-16 weeks old) C57BL/6 Ck1ε^Tau/Tau^ male mice kept in light-tight chambers with food and water available ad libitum. Mice were kept for > 7 days in 10:10 LD schedule, before being released in constant red dim light for ≥ 10 days. All animal work was conducted and licensed in accordance with the Code of Practice for the Housing and Care of Animals Bred, Supplied or Used for Scientific Purposes under A(SP)A and the EU Directive 2010/63/EU, and with local ethical approval (MRC-LMB AWERB).

#### Transgenic animal models

Grin2C inducible Cre knock-in mice were generated by the Wellcome Trust Sanger Institute (allele: *Grin2*^*tm1CreERT2_EGFP/Wtsi*^*)*. PER2::LUC mice (allele: *B6.129S6-Per2tm1Jt/J*) were gifted by Joe Takahashi (UT Southwestern, US); Ck1ε Tau mice (allele: *B6.129-Csnk1etm1Asil/J*) were gifted by Andrew Loudon (University Manchester, UK).

### Method Details

#### Experimental Design

All ex vivo and in vivo experiments were performed on at least three animals. In vivo experiments were performed on two independent cohorts of mice. Number of experimental replicates (n) is indicated in figure legend and text and refers to the number of animals used independently treated in each experimental conditions, whereas N refers to the number of cells within an SCN slice/section. Animals were selected in an unblended manner, but no specific randomization strategy was used. Statistical computations were not performed to determine the optimal sample-size for experiments. Data from all the experiments were included in the analysis, with the only exclusion of SCN slices/animals being those that died for unrelated technical reasons (e.g., inadequate seal of the glass cover on petri dishes for ex vivo experiments, or mouse death caused by surgical complications in vivo).

#### AAV transduction of SCN slices

SCN organotypic slices from p12-p14 mice were obtained, cultured and transduced as previously described (Brancaccio et al., 2013). AAVs encoding Syn-RCaMP1h, Syn-iGluSnFR, GFAP-iGluSnFR, (Loren L. Looger and the HHMI Janelia Farm Research Campus), Syn-ArcLightD (Vincent A. Pieribone), gfaABC_1_D-GCaMP3, gfaABC_1_D-LCK::GCaMP6 (Baljit S. Khakh), CMV.PI.Synaptophysin::GCaMP3.SV40 (Leon Lagnado), Syn-EGFP, GFAP-EGFP, EF1α-flex-EYFP were purchased from Penn Vector Core. AAVs encoding GFAP-hM3DGq:mCherry (Bryan Roth), and GFAP-mCherry::Cre and Syn-mCherry:Cre, GFAP-EGFP:Cre, EF1α-flex-taCasp3-TEVp (Nirao Shah) were purchased from UNC Gene Therapy Center Vector Core. For neuronal or astrocyte-selective Tau knock-out experiments, bioluminescence from Ck1ε^Tau/Tau^ SCN slices expressing PER2::LUC was recorded for ≥ 5 days (Before Cre) in medium containing 100 μM luciferin (Promega). Slices were briefly taken out from the PMT tubes for AAV transduction (Syn-mCherry::Cre or GFAP-mCherry:Cre) and immediately returned for PMT recording, with no medium change. Dynamic changes in PER2::LUC signal were recorded in real-time for ≥ 10 days; no further treatment was performed. No phenotype was generally observed during the first 4 days post-transduction (0-4 dpt), consistent with time required for AAV infection cycle (Edwards et al., 2016). After that, a phenotype became evident and was assessed from day 4 to day 8 post-transduction (4-8 dpt). Transduction efficiency was verified at the end of the experiment by assessing number of mCherry^+^ cells/SCN area. For localization of the NR2C subunit in the SCN Grin2C inducible Cre knock-in mice were imported from the Wellcome Trust Sanger Institute (allele: *Grin2*^*tm1CreERT2_EGFP/Wtsi*^). Dorsal expression of the EGFP tag was confirmed by confocal microscopy, but was under the detection limit of the LV200 Olympus units used for in vivo long-term imaging. In order to localize the NR2C expressing neurons for live imaging experiments SCN slices were transduced with CAG-flex-tdTomato AAV vectors. A week after transduction Cre dependent expression of the tdTomato reporter was induced by incubating SCN slices with (Z)-4-OH-tamoxifen (10 μM) for 2 days, before washing out the drug and starting the DQP-1105 experiments. A strong fluorescent signal became apparent 2 days after tamoxifen induction in SCN slices from heterozygote mice and absent in co-treated wild-type littermates, used as control.

#### Drug treatments

DL-TBOA, UCPH-101, WAY 213613, DQP-1105, QNZ-46, TCN-201, (Z)-4-OH-tamoxifen DNQX, MK-801 hydrogen maleate were purchased from Tocris Bioscience; MSO from Sigma-Aldrich. All drugs were diluted in DMSO or ddH2O as required for treatment and corresponding concentration of the dissolving agent used as vehicle controls. For washout, slices were washed 3 times and transferred to fresh medium containing luciferin. SCN slices were kept in standard culture medium (DMEM based with 5% serum and Glutamax supplement 1X (Life Technologies), unless otherwise specified (see main text).

#### Immunofluorescence on SCN slices

Antiserum for immunofluorescence on SCN slices: goat anti-glutamate transporter neuronal EAAT3 Millipore 1:1000; rabbit anti-GAD65/67 1:1000 Abcam; rabbit anti-AVP, Bachem 1:500; rabbit anti-Aldh1L1 1:200 Abcam; rabbit anti-NG2 1:200 Abcam; goat anti-Iba1 1:350 Wako; Secondary antibodies: donkey anti-goat conjugated with Alexa 647; donkey anti-goat conjugated with Alexa 488; chicken anti-rabbit conjugated with Alexa 488; goat anti-rabbit conjugated with Alexa 488; goat anti-rabbit conjugated with Alexa 647 (Life Technologies). Targeting rates and co-localization analysis of Syn-mCherry::Cre and GFAP-EGFP::Cre co-transduced SCN slice was manually performed on single confocal planes. Number of DAPI^+^ cells was also assessed manually, because of natural unevenness of DAPI staining in thick slices. Co-localization of GFAP-mCherry::Cre with the different astroglial markers (Aldh1L1, Iba1, NG2) was also manually performed on single confocal planes.

#### Multi-channel long-term live-imaging

Multi-channel bioluminescence/fluorescence imaging was generally performed as previously described (Brancaccio et al., 2013), using the LV200 system (Olympus Microscopy, UK). Exposure varied between 200-600ms for fluorescent reporters, bioluminescence signal was acquired over 30 min. Time resolution is 30 min. For imaging of neuronal- or astrocyte-selective Tau knock-out experiments, *Ck1ε^Tau/Tau^* SCN expressing PER2::LUC and previously co-transduced with Syn-RCaMP1h and gfaABC_1_D-GCaMP3 were super-transduced with GFAP-mCherry::Cre and Syn-mCherry::Cre and immediately put on camera. Beginning of the recording corresponds to the “0-4 dpt” stage in PMT experiments. No further procedures were performed and time series in different fluorescence and bioluminescence channels were used as single time-series stacks. Image analysis and Center of Luminescence (CoL) were performed as previously described (Brancaccio et al., 2013). The Igor Pro (Wavemetric) plugin SARFIA (Dorostkar et al., 2010, Zielinski et al., 2014) was used for semi-automatic image analysis at single-cell levels. To compare CoL data across different SCN slices, x and y coordinates of CoL were normalized and the mean value of CoL trajectories over 3 days calculated. The calculation was performed before and in the presence of the drug for each sample. For the temporal misalignment experiment, data from the first 3 days of recording were pooled (“0-4 dpt” stage, no effect) and compared with the last 3 days of the “4 to 8 dpt” phase from neuronal- and astrocytic-Ck1ε^Tau/Tau^ -deleted slices.

#### Electrophysiological recordings

Organotypic SCN slices were phase-mapped by using PER2::LUC reporter recorded by PMTs. Slices were cut from their Millipore insert and transferred to a bath continuously perfused with recording solution (in mM): 125 NaCl, 25 NaHCO3, 3 KCl, 1.25 NaH2PO4, 25 Glucose, 2 CaCl2 and 1 MgCl2, gassed with 95% O2 and 5% CO2 and heated to 33°C to 35°C. Recordings commenced one hour later and were preferentially targeted to the dorsomedial SCN, as assessed by visual inspection. Pipettes (5-8 MΩ) were filled with the following intracellular solution (in mM): 135 K-gluconate, 7 NaCl, 10 HEPES, 2 Na2-ATP, 0.3 Na2-GTP, 2 MgCl2, 0.01 biocytin, pH 7.2 to 7.3, with KOH. Whole cell current clamp recordings were made with a Cornerstone BVC-700A amplifier (Dagan) and were completed within 2-5 min of membrane rupture to minimize the effects of dialysis of the cells. DQP-1105 (50μM) or 0.1% DMSO (vehicle) was applied to the slice by local pressurized perfusion via a quartz micromanifold (ALA Scientific Instruments) placed close to the recording site. Drugs were diluted from stock into the recording solution and warmed before pressure application (10-15psi). Drug delivery was controlled by the acquisition software. Serial recordings were made, where vehicle was applied to the cells followed by washout and drug application. Before another recording was made from the same slice, the micromanifold was manually flushed with recording solution and the slice was left for 15 min to allow the drug to be completely washed out of the bath. Signals were digitized at 10kHz using Axograph X (Axograph Scientific). Data were analyzed using the Neuromatic plugin (Jason Rothman, University College London; see http://neuromatic.thinkrandom.com) for Igor Pro (WaveMetrics). Recordings were liquid junction potential corrected a posteriori and membrane potential was determined by fitting a Gaussian distribution to a frequency plot of the analysis window. Depolarization data were presented as the difference between the depolarization caused by vehicle application and the depolarization caused by drug application for each recording. Statistical analysis was performed using unpaired Student’s t test and all electrophysiological data were determined to be parametric by a Kolmogorov-Smirnov normality test.

#### In vivo deletion of Ck1ε^Tau/Tau^ in SCN neurons or astrocytes

Wheel-running behavior was recorded in individually caged (8-16 weeks old) C57BL/6 *Ck1ε^Tau/Tau^* male mice kept in light-tight chambers with food and water available ad libitum. Mice were kept for > 7 days in 10:10 LD schedule, before being released in constant red dim light for ≥ 10 days. AAV encoding for GFAP-mCherry::Cre, Syn-mCherry::Cre, or GFAP-EGFP and Syn-EGFP controls were stereotaxically injected as previously described (Edwards et al., 2016). Mice were left in 10:10 LD conditions for recovery for 10-15 days after surgery before being released in DD for period determination. Period measured by Lomb-Scargle periodogram in ClockLab6 (ActiMetrics). Effective SCN targeting was confirmed ex post by expression of mCherry or EGFP tags with a fluorescent HCA microscope, equipped with a 4X objective (Nikon). Neuronal or astrocytic identity of targeted cells confirmed by confocal imaging and co-localization of mCherry marker with GFAP (1:1000, chicken polyclonal, Abcam) and Aldh1L1 (1:400, rabbit polyclonal, Abcam) antisera. Co-localization analysis of Syn-mCherry::Cre and GFAP-mCherry::Cre with GFAP was performed manually on confocal single planes. 1-2 confocal planes were taken into account from at least two separate sections on the antero-posterior brain axis in each single animal. The procedure was repeated on three animals. Number of DAPI^+^ cells was assessed by the semi-automated nucleus counter plugin of the Fiji Cookbook.

### Quantification and Statistical Analysis

Data analyses of period, amplitude and relative amplitude error (RAE) were generally performed by using the FFT-NLLS function of the online BioDare suite (Moore et al., 2014, Zielinski et al., 2014) (https://www.biodare.ed.ac.uk) (Courtesy of Prof. Andrew Millar, University of Edinburgh). In all the experiments n indicates the number of independent animals/slices analyzed. This information is explicitly reported in figure legend/ texts for each experimental group. For ex vivo experiments each biological replicate is a single SCN slice coming from a single mouse. For in vivo experiments each biological replicate is single animal. “N” refers to the number of cells within a single SCN slice for ex vivo experiments using bioluminescence and fluorescence live recording. For immunofluorescence studies on fixed brain sections and slices, “N” represents the total number of cells counted over 3 or more experimental replicates. Overall peak phase for landscape analysis in Figure 1 was assessed by manual inspection. Because of the prolonged flat peak (“anti-pulse”) of the waveform of the iGluSnFR and gfaABC1D-GCaMP3 reporters the midline of the fluorescence rise was used, instead, to assess the peak phase. Phase detection of single cellular oscillations in population studies was assessed in BioDare. Anti-pulse waveforms of iGluSnFR and gfaABC_1_D-GCaMP3 did not allow reliable automated detection of phases of cellular oscillations in BioDare. In such cases the peak of the inverted waveform was used (trough of the original wave) to project the peak of the original waveform, normalized for period (Figure 2F). This procedure was confirmed to assess phases correctly and reliably by comparing results with manually detected phases on the same data sample. For period scatter analysis of cellular oscillations within a single slice, median and interquartile range (IQR) as measure of variance were considered and significance assessed by Kolmogorov-Smirnov test, because data were not independent and not normally distributed. For comparison across different SCN slices, mean value of the medians and the SEM of the IQR were considered instead and statistical significance assessed with 2-way ANOVA for Repeated-measures (RM), with a Bonferroni correction for multiple comparison in Prism 6 (GraphPad). Amplitude and RAE ratio were compared by ANOVA. Circular statistics to assemble Raleigh plots and compute mean vector lengths for landscape and phase dispersal analysis was performed by using Oriana 4 (Kovacs Computer Services, UK). For skewness analysis waveforms of > 100 cellular oscillations in each channel (Syn-RCaMP1h^+^, PER2::LUC^+^, Syn-iGluSnFR^+^) were followed. Waveforms of single oscillations on the day before DL-TBOA, the last day in the presence of DL-TBOA and the last day of the washout were used to construct mean traces. Point 0 was the trough of iGluSnFR signal, coincident with the RCaMP1h peak, given the antiphasic nature of the two rhythms. In the case of PER2::LUC waveform analysis, the trough of the PER2::LUC oscillation on the same day was registered to the point 0, to compensate for the different phase. Skewness of the mean traces was assessed in Prism. The analysis was repeated across 3 slices and their mean skewness compared by 2-way ANOVA, Bonferroni corrected.

### Data and Software Availability

All the analyses in this manuscript have been conducted with commercially available or free software.

## Author Contributions

M.B. designed, performed, and analyzed the experiments except for electrophysiological recordings performed and analyzed by A.P.P. M.H.H. contributed to the experimental design. E.S.M. and J.E.C. conducted preliminary experiments. All authors contributed to project discussions. M.B. and M.H.H. wrote the manuscript.

## Supplemental Information

Document S1. Figures S1–S8

Movie S1. Anti-phasic Rhythms of Neuronal [Ca^2+^]_i_ and Extracellular [Glu]_e_ in the Suprachiasmatic Nucleus Related to Figure 1. Long-term fluorescent live imaging of SCN slices co-transduced with AAVs expressing the RCaMP1h or iGluSnFR to detect neuronal intracellular calcium and extracellular glutamate, respectively. Co-detection of these reporters revealed that sustained widespread anti-phasic oscillations [Ca^2+^]_i_ and [Glu]_e_ are present in the SCN.

Movie S2. CoL Analysis of PER2::LUC Spatiotemporal Wave in SCN Slices Treated with the NR2C Inhibitor DQP-1105 Related to Figure 4. SCN slices expressing PER2::LUC were treated with the NR2C antagonist DQP-1105. Following drug addition, the original daily trajectory of the CoL of the bioluminescent distribution (black contour) shifted to a more ventral position (white contour), coherently with the dorsal expression of the NR2C subunit.

Movie S3. CoL Analysis of PER2::LUC Spatiotemporal Wave in Astrocytically Restricted Ck1ε^Tau/Tau^ Knockout Related to Figure 7. SCN slices expressing PER2::LUC from floxed Ck1ε^Tau/Tau^ mice were transduced with AAVs encoding Cre recombinase, driven by the GFAP promoter. Real-time bioluminescent recording started immediately after AAV transduction to follow the dynamic of the spatiotemporal changes in PER2::LUC expression upon astrocytic knockout of the Tau allele. After a lag, due to viral transduction cycle, the original daily trajectory of the CoL of the bioluminescent distribution (white contour) changed its shape and dynamically shifted to a more dorsal stable position (black contour). Therefore, re-programming of TTFL clock in astrocytes re-programmed the spatiotemporal wave of clock gene expression of the SCN to a new and specific state.

Document S2. Article plus Supplemental Information
