# Supplementary material for: Astrocytes Control Circadian Timekeeping in the Suprachiasmatic Nucleus via Glutamatergic Signaling
Source: Neuron. 2017 Mar 22;93(6):1420–1435.e5. doi: 10.1016/j.neuron.2017.02.030 (PMC5376383; doi:10.1016/j.neuron.2017.02.030)
Supplement: Document S1. Figures S1–S8 [file mmc1.pdf]

Neuron, Volume *93*

# Supplemental Information

# Astrocytes Control Circadian Timekeeping in the Suprachiasmatic Nucleus via Glutamatergic Signaling

**Marco Brancaccio, Andrew P. Patton, Johanna E. Chesham, Elizabeth S. Maywood, and Michael H. Hastings**

**Supplemental Information**

**Supplemental Figures**

**Figure S1**

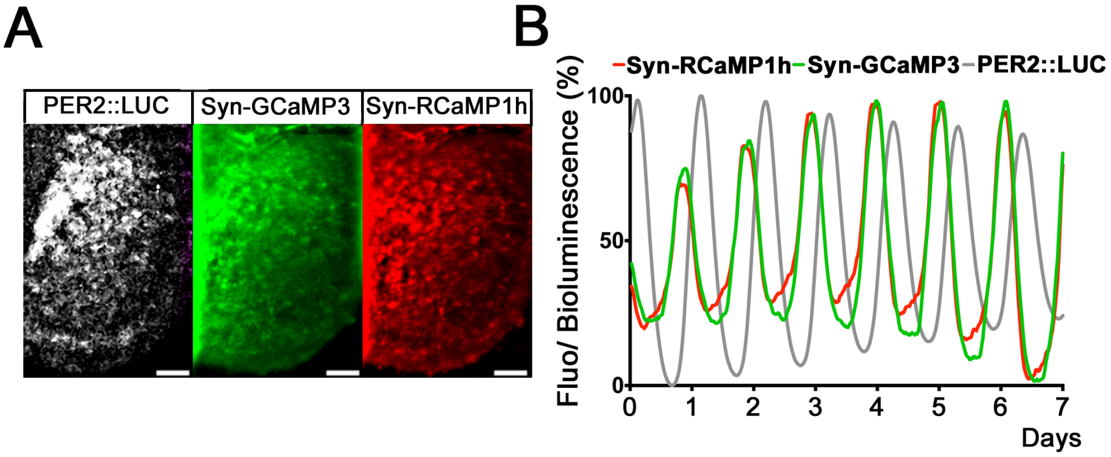


**Figure S1: Validation of Syn-RCaMP1h against Syn-GCaMP3 for detection of neuronal $[Ca^{2+}]_i$ circadian oscillations.** Related to Figure 1

(A) Microphotographs of PER2::LUC SCN slices simultaneously transduced with AAVs expressing Syn-RCaMP1h and Syn-GCaMP3, both driven by the Syn promoter (B) Representative traces of simultaneously detected PER2::LUC bioluminescence and RCaMP1h/GCaMP3 fluorescence to compare peak-phases of the different reporters. Scale Bar= 50 µm.

**Figure S2**

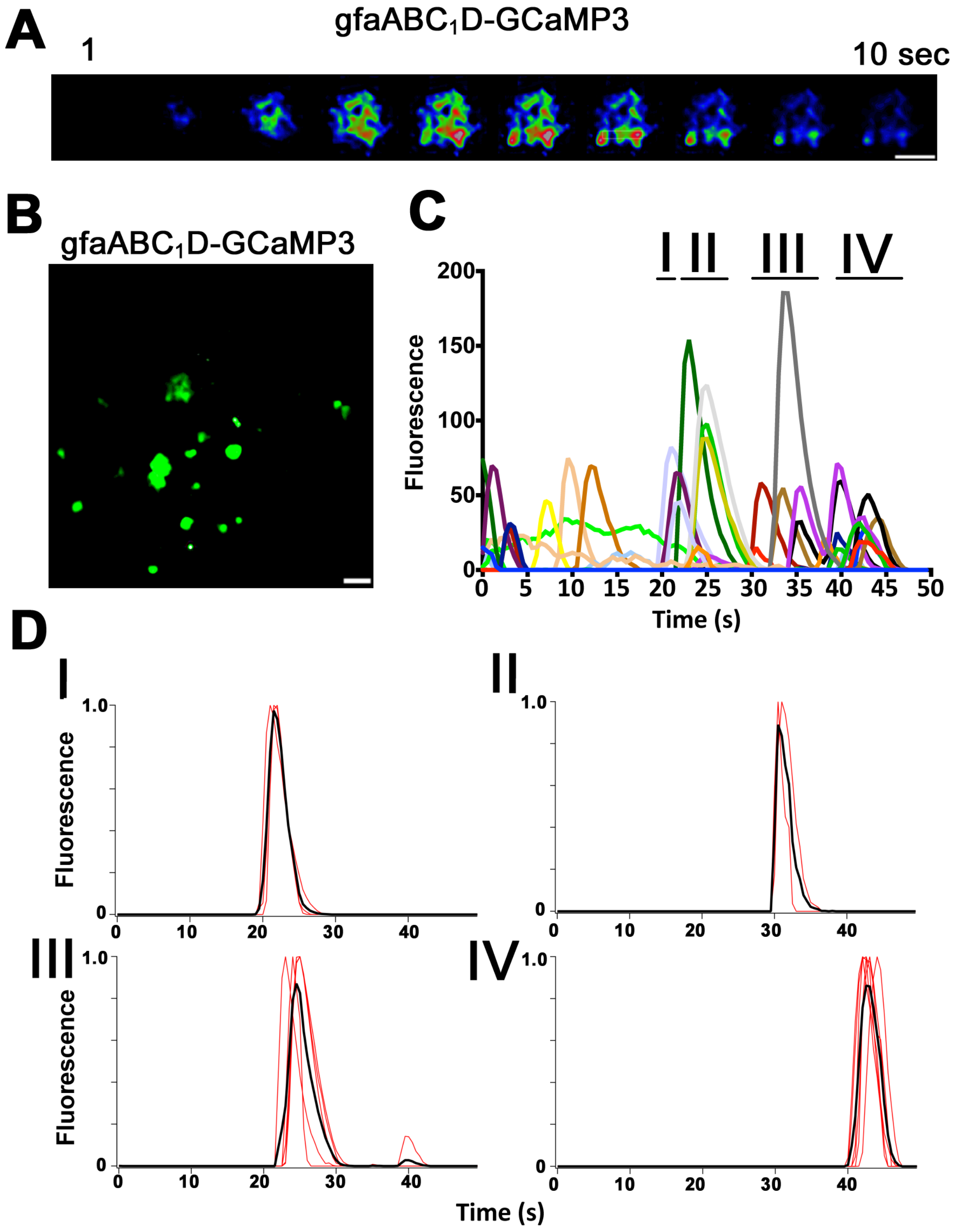


**Figure S2. Fast waves of intracellular calcium detected in astrocytes by the gfaABC$_1$D-GCaMP3 reporter.** Related to Figure 1

(A) Fluorescent signal (LUT: rainbow) from a representative astrocyte from SCN slice transduced with AAV encoding the calcium sensor GCaMP3 driven by the gfaABC$_1$D promoter. (B) Microphotograph still showing several rapid bursts of astrocytic calcium in SCN, reported by gfaABC$_1$D-GCaMP3. (C, D) Overall representation and clustering (groups I to IV) of astrocytic calcium bursts). Scale Bar: 15 µm.

**Figure S3**

**Figure S3**

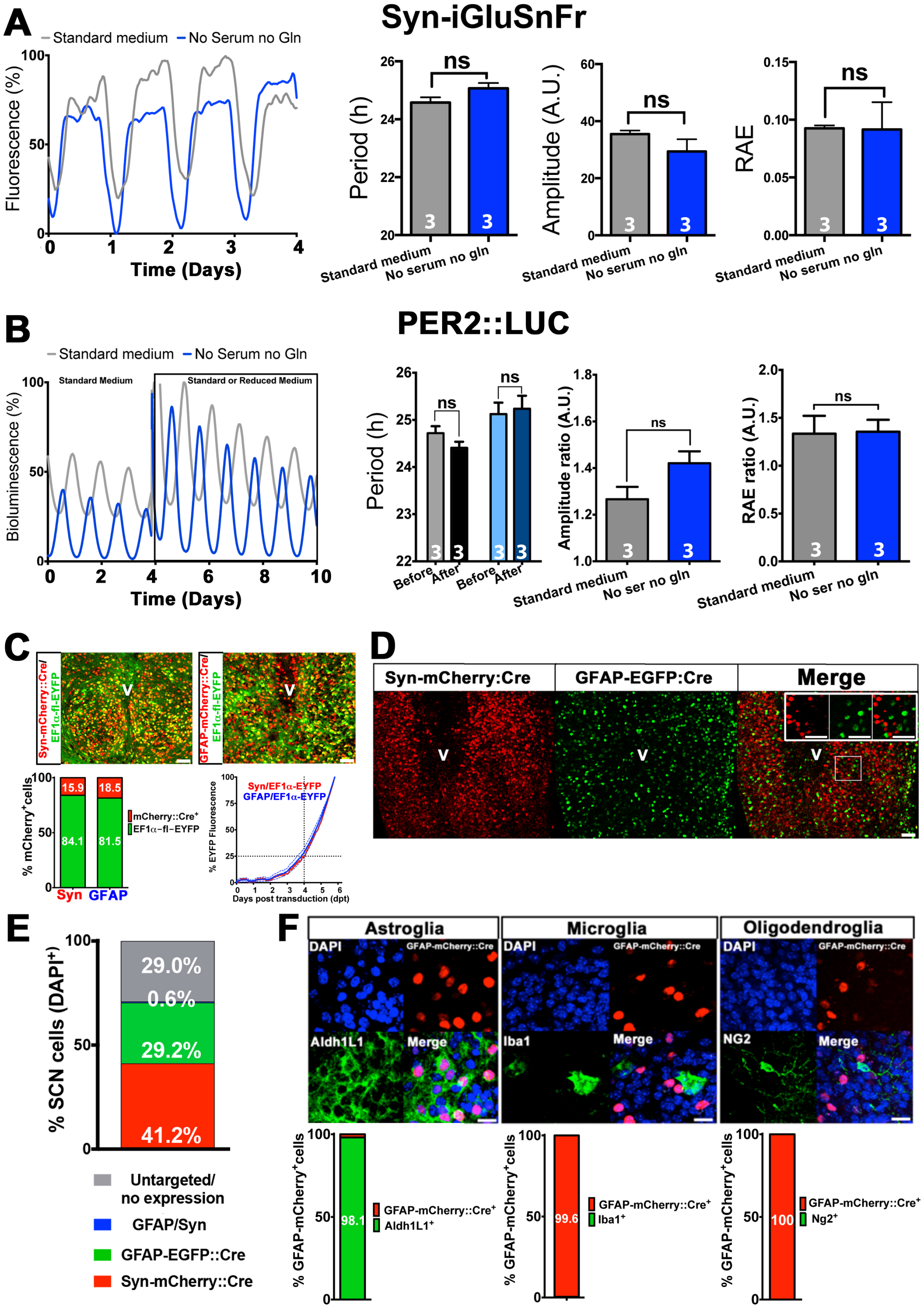

**Figure S3. Efficiency and specificity of *ex vivo* targeting of astrocytes in SCN slices by AAVs expressing GFAP-mCherry::Cre.** Related to Figure 1**.**

(A, B) Representative traces from SCN slices transduced by Syn-iGluSnFR or expressing the PER2::LUC reporter kept in standard medium (5% serum, Gln supplement, *gray lines)* or Gln- and serum-deprived conditions (*blue lines*). (C) Representative confocal micrographs and co-expression rates of SCN slices expressing GFAP-mCherry::Cre and Syn-mCherry::Cre co-transduced with a flexed EYFP expressing AAV reporter (EF1α-flex-EYFP) to estimate efficiency and timing of Cre recombination ($N_{Syn\text{-}mCherry::Cre}^{+}$=1846; n=2; $N_{GFAP\text{-}mCherry::Cre}^{+}$=1696; n=2; Temporal traces are Mean±SEM, n=2). (D) Tiled confocal micrographs of SCN slices co-transduced with GFAP-EGFP::Cre and Syn-mCherry::Cre. Inset: magnification of boxed area showing no co-localization of the fluorescent tags. Scale Bar= 50µm. (E) Stacked bar plot representing percentage of Syn-mCherry::Cre and GFAP-EGFP::Cre in SCN transduced slices and co-localization rates of the two reporters ($N_{DAPI}^{+}$= 2278, n=3. (F) Confocal micrographs and co-localization analysis of SCN transduced with GFAP-mCherry::Cre$^{+}$ and co-stained with astroglial lineage markers. As expected, Aldh1L1 is highly co-expressed in GFAP-mCherry::Cre$^{+}$ cells ($N_{GFAP\text{-}mCherry::Cre+}$ =540; n=3), whereas microglial Iba1 $N_{GFAP\text{-}mCherry::Cre}^{+}$ = 1528;n=3), or oligodendroglial NG2 markers ($N_{GFAP\text{-}mCherry::Cre}^{+}$=808; n=3) do not show any expression in GFAP-targeted cells. Scale bar: 20µm. All bar graphs are Mean±SEM; n experimental replicates depicted on bars. Statistical test for period is 2-way RM-ANOVA, Bonferroni corrected. Statistical test for amplitude and RAE ratio is 1-way ANOVA, Bonferroni corrected.

**Figure S4**

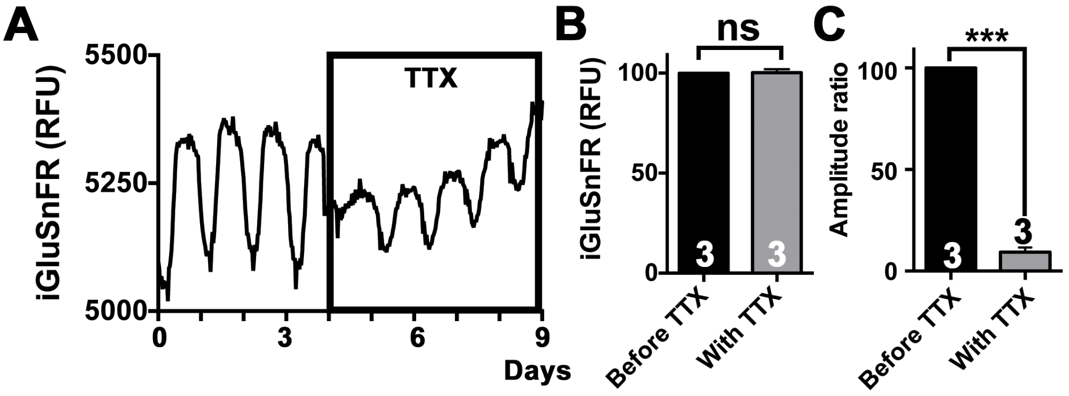


**Figure S4. Suppression of synaptic activity by TTX does not abolish endogenous rhythms of extracellular glutamate in SCN slices.** Related to Figure 1

(A) Representative trace from SCN slices expressing Syn-iGluSnFR to detect extracellular glutamate before TTX treatment and in the presence of the drug. (B, C) Bar graphs showing baseline fluorescence and circadian amplitude ratio before TTX treatment and in the presence of the drug (C) All bar graphs are Mean±SEM, n experimental replicates depicted on bars. Statistical test: 2-tailed paired t-test ***p<0.001

**Figure S5**

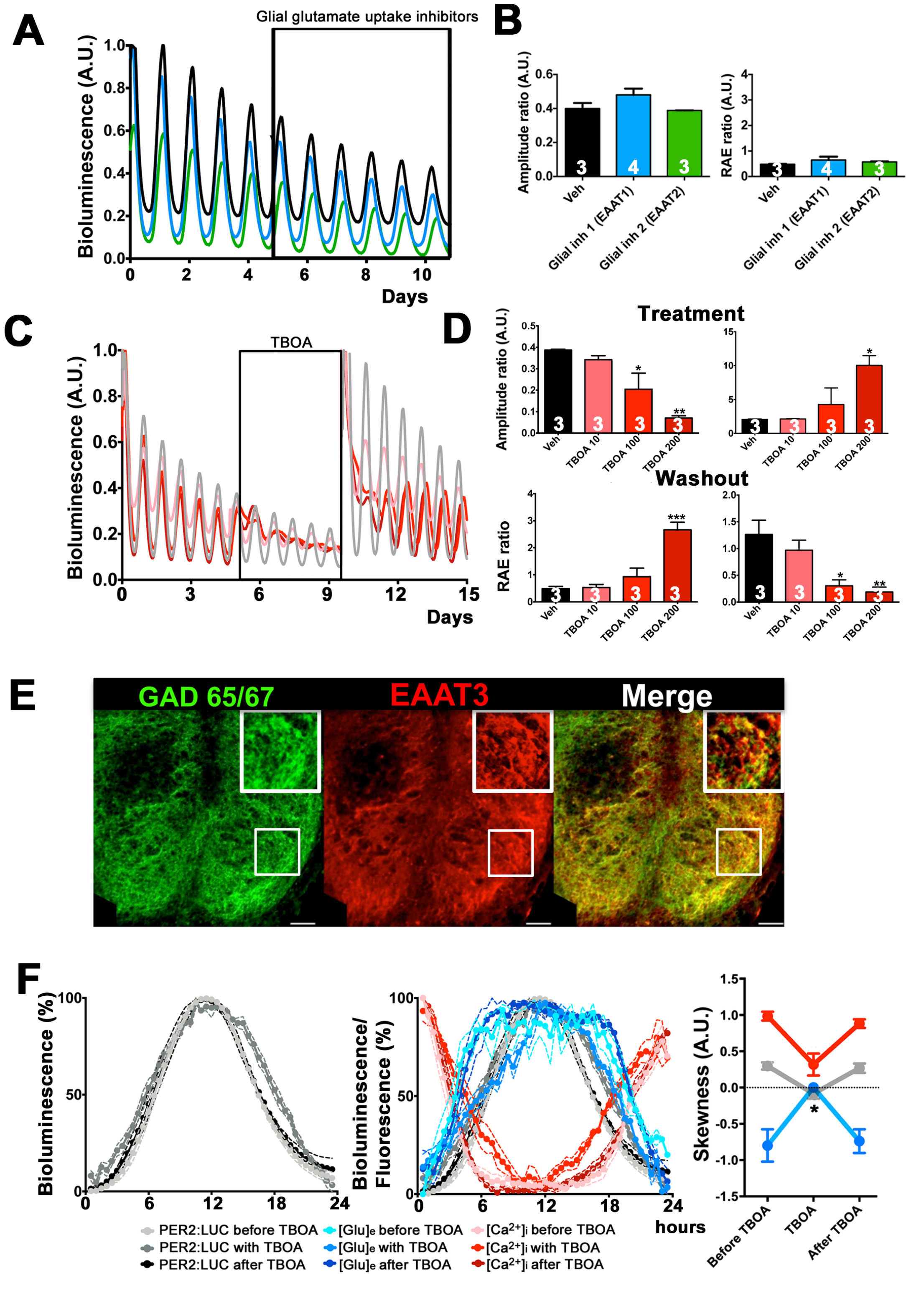


**Figure S5. Effects of glutamate uptake inhibitors on circadian oscillations.** Related to Figure 2

(A, B) Representative traces and bar graphs of amplitude or robustness (RAE) of the PER2::LUC oscillations in SCN slices treated with blockers of the glial isoforms of glutamate transporters EAAT1 (UCPH-101 20 µM, *blue lines*)

or EAAT2 (WAY 213613 10 µM, *green lines*), or vehicle *(black lines)*. (C) Representative traces of PER2::LUC expressing SCN slices challenged with increasing concentrations of DL-TBOA. (D) Dose-response curve of amplitude and RAE ratio PER2::LUC oscillations in the presence of increasing concentrations of DL-TBOA (10, 100, 200 µM- *shades of red*), All bar graphs are Mean±SEM; n experimental replicates depicted on bars. Statistical test for amplitude and RAE ratio is 1-way ANOVA, Bonferroni corrected. (E) Representative photomicrographs of SCN slices stained with anti-GAD 65/67 *(green channel)* and anti EAAT3 *(red channel)* anti-sera. Inset: EAAT3 expression in GAD 65/67$^{+}$ neurons. (F) Super-imposed Mean±SEM waveforms of PER2::LUC bioluminescence and RCaMP1h and iGluSnFR fluorescence before DL-TBOA treatment, in the presence of the drug and after washout. $[Glu]_e$ and $[Ca^{2+}]_i$, traces are re-plotted from main text for comparison *=p<0.05; **p<0.01; ***p<0.001; 2-way RM-ANOVA, Bonferroni corrected, n≥3. Scale Bar= 50 µm.

**Figure S6**

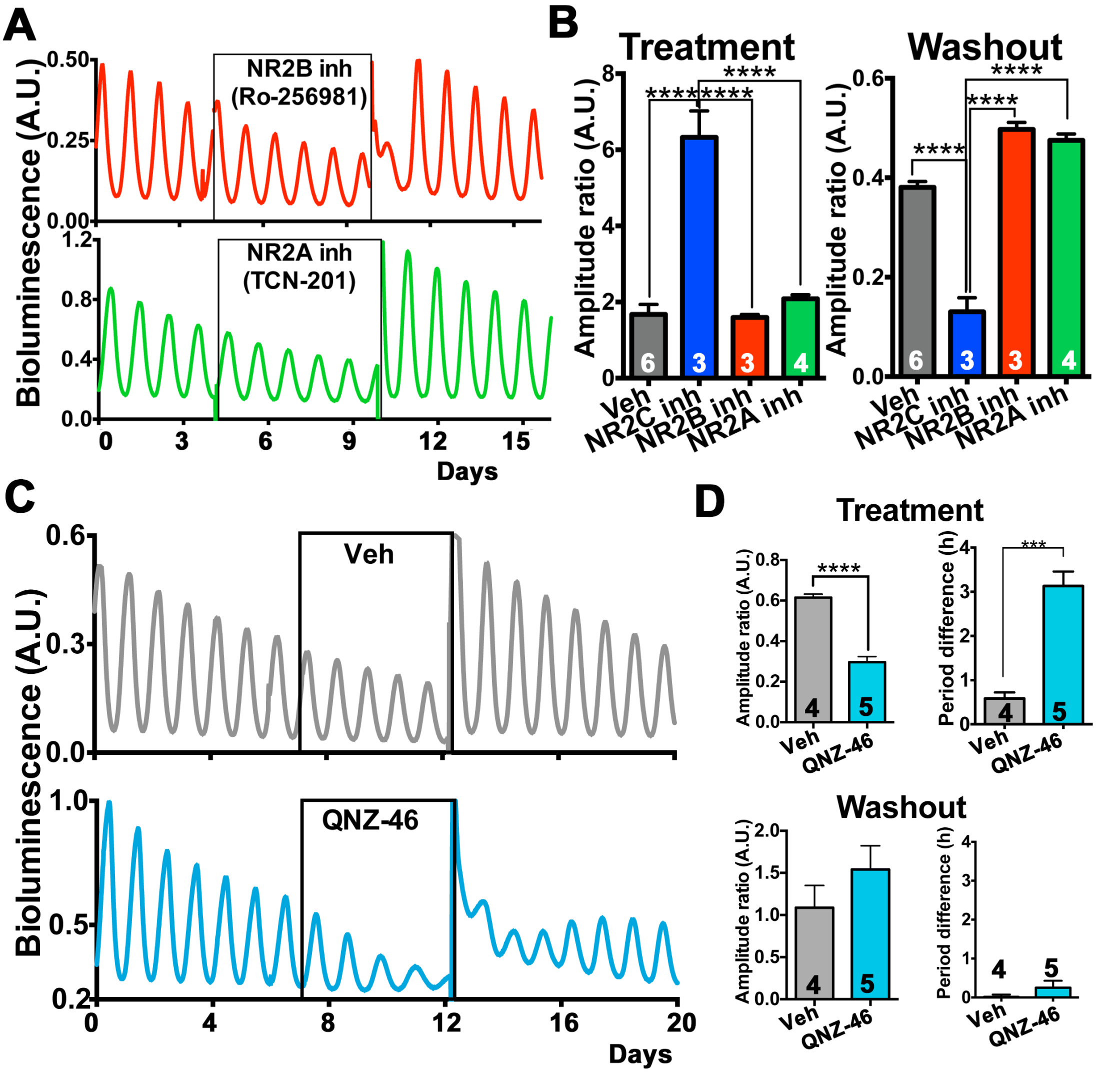


**Figure S6: Selective antagonism of the NR2C subunit of the NMDA receptor damps circadian oscillations of PER2::LUC and lengthens their period.** Related to Figure 3

(A) Representative traces from SCN slices expressing PER2::LUC and treated with competitive antagonists selective for NR2A and B subunits of the NMDA receptor. (B) Bar graphs showing amplitude of PER2::LUC oscillations in SCN slices treated with NMDAR subunit specific antagonists, in the presence of the drug and after washout (*treatment data* are re-plotted from Figure 3 for comparison). (C) Representative traces from SCN slices expressing PER2::LUC and treated with the NR2C selective competitive antagonist QNZ-46 (20µM) or vehicle. (D) Bar graphs showing amplitude and period of PER2::LUC$^{+}$ SCN slices upon QNZ-46 treatment and after drug washout. All bar graphs are Mean±SEM; n experimental replicates depicted on bars. Statistical test: 1-way ANOVA, Bonferroni corrected; ***p<0.001; ****=p<0.0001

**Figure S7**

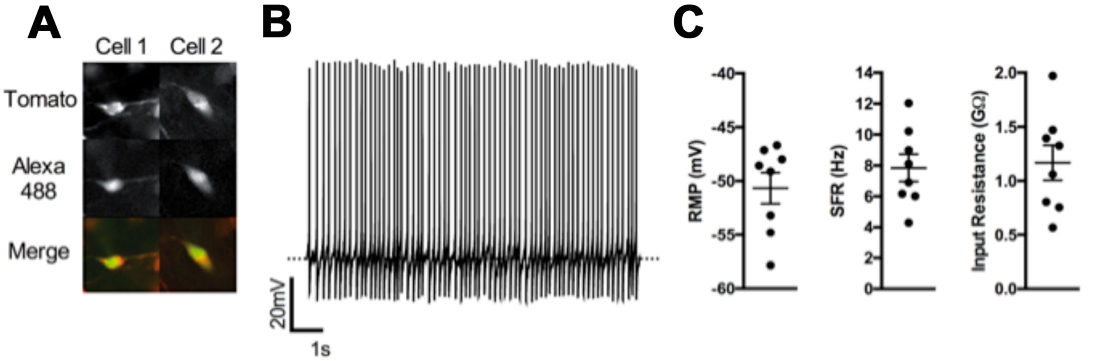


**Figure S7: Electrophysiological properties of Grin2C-tdTomato$^+$ neurons.** Related to Figure 4

(A) Representative microphotographs of biocytin-Alexa488 filled GRIN2C tdTomato$^+$ cells. (B) Example of whole cell current clamp recording showing spontaneous action potentials and resting membrane potential. The dashed line indicates -55mV. (C) Summary of basic electrophysiological properties of Grin2C-tdTomato$^+$ neurons (dots indicate individual cells, lines are Mean± SEM; n=8. RMP is resting membrane potential SFR is spontaneous firing rate.

Figure S8

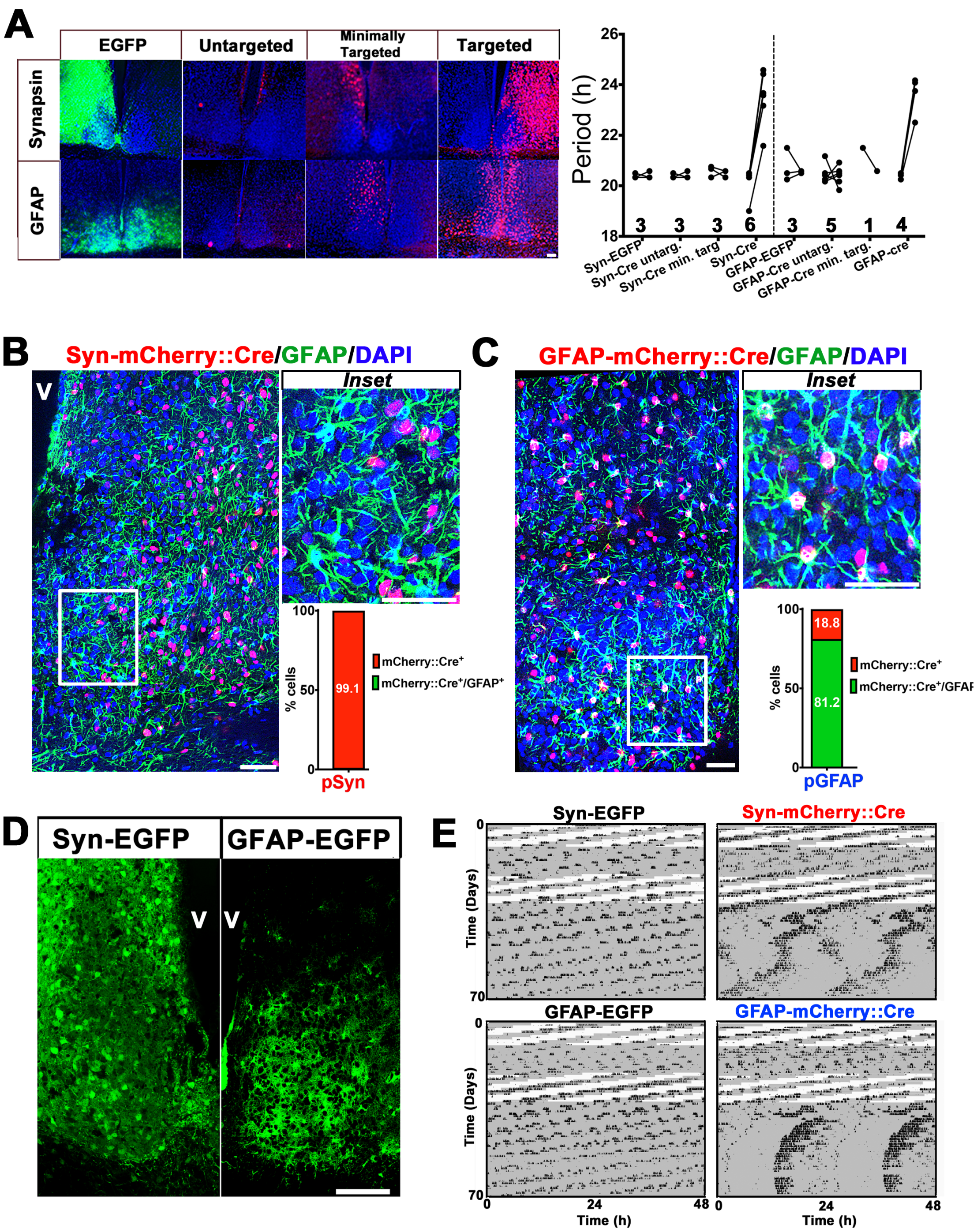


**Figure S8: Stereotaxic targeting of neuronal and astrocytic populations *in vivo* by Syn and GFAP promoters.** Related to Figure 8

(A) Photomicrographs of SCN sections from mice stereotaxically targeted with AAVs expressing respectively mCherry::Cre or EGFP under control of Syn or GFAP promoters, showing correspondence between levels of AAV targeting and behavioural effects on period. Data relative to targeted mice mCherry/EGFP mice are re-plotted from Figure 8 to show internal variation of

period in individual mice. Numbers in the figure are n experimental replicates for each subgroup following ex post histological assessment of targeting. (B, C) Confocal photomicrographs and co-localization rates of mCherry::Cre$^{+}$ cells with the astrocytic marker GFAP in SCN sections targeted with: Syn-mCherry::Cre (B) and GFAP-mCherry::Cre (C), respectively ($N_{GFAP\text{-}mCherry::Cre}$=977; n=3 $N_{Syn\text{-}mCherry::Cre}$=2630; n=3). (D) Confocal micrographs of SCN from mice targeted with EGFP tag driven by Syn or GFAP promoter show distinct neuronal and astrocytic morphologies, respectively. Scale bars= 50 µM. V indicates 3$^{rd}$ ventricle. (E) Actograms of mice presented in Figure 8A, re-plotted on a 24h scale.

**Supplemental Movies**

**Movie S1 still**

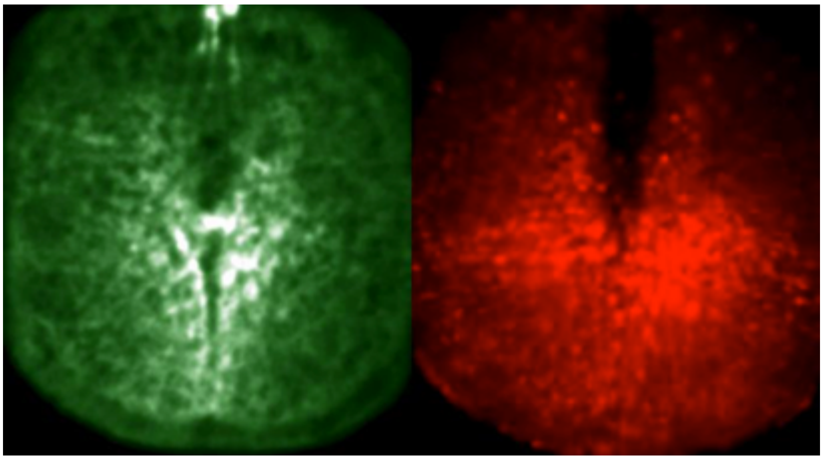

**Movie S1: Anti-phasic rhythms of neuronal $[Ca^{2+}]_i$ and extracellular $[Glu]_e$ in the suprachiasmatic nucleus.** Related to Figure 1

Long-term fluorescent live imaging of SCN slices co-transduced with AAVs expressing the RCaMP1h or iGluSnFR to detect neuronal intracellular calcium and extracellular glutamate, respectively. Co-detection of these reporters revealed that sustained widespread anti-phasic oscillations $[Ca^{2+}]_i$ and $[Glu]_e$ are present in the SCN.

**Movie S2 still**

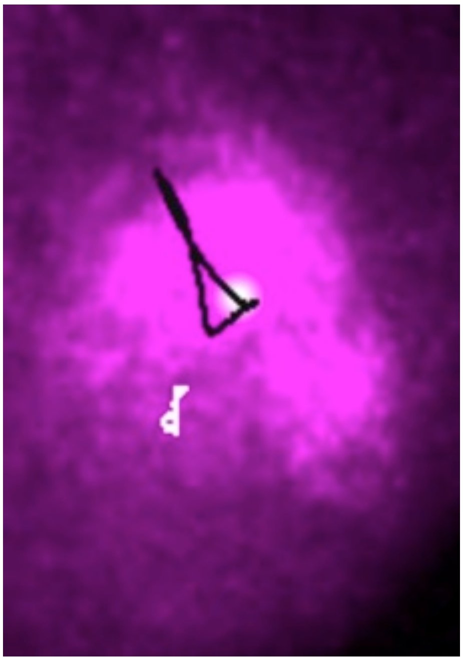

**Movie S2: CoL analysis of PER2::LUC spatio-temporal wave in SCN slices treated with the NR2C inhibitor DQP-1105.** Related to Figure 4

SCN slices expressing PER2::LUC were treated with the NR2C antagonist DQP-1105. Following drug addition the original daily trajectory of the CoL of the bioluminescent distribution *(black contour)* shifted to a more ventral position *(white contour)*, coherently with the dorsal expression of the NR2C subunit.

**Movie S3 still**

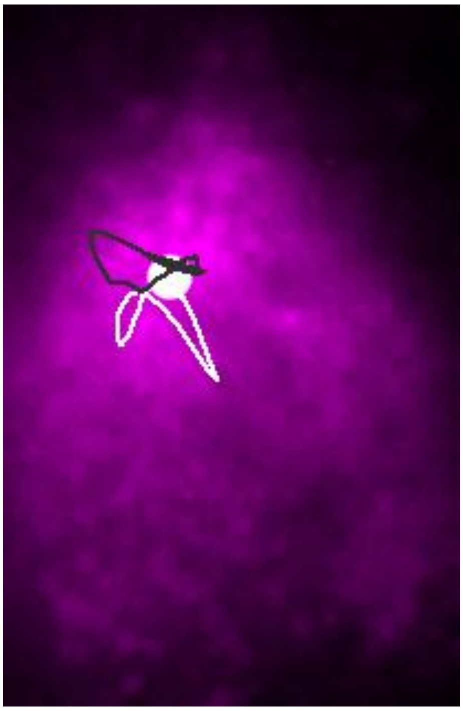

**Movie S3: CoL analysis of PER2::LUC spatio-temporal wave in astrocytically restricted Ck1ε$^{Tau/Tau}$ knock-out.** Related to Figure 7

SCN slices expressing PER2::LUC from floxed Ck1ε$^{Tau/Tau}$ mice were transduced with AAVs encoding Cre recombinase, driven by the GFAP promoter. Real-time bioluminescent recording started immediately after AAV transduction to follow the dynamic of the spatio-temporal changes in PER2::LUC expression upon astrocytic knock-out of the Tau allele. After a lag, due to viral transduction cycle, the original daily trajectory of the CoL of the bioluminescent distribution *(white contour)* changed its shape and dynamically shifted to a more dorsal stable position *(black contour)*. Therefore, re-programming of TTFL clock in astrocytes re-programmed the spatio-temporal wave of clock gene expression of the SCN to a new and specific state.
