# Supplementary figures and images for: Astrocytes Control Circadian Timekeeping in the Suprachiasmatic Nucleus via Glutamatergic Signaling
Source: Neuron. 2017 Mar 22;93(6):1420–1435.e5. doi: 10.1016/j.neuron.2017.02.030 (PMC5376383; doi:10.1016/j.neuron.2017.02.030)

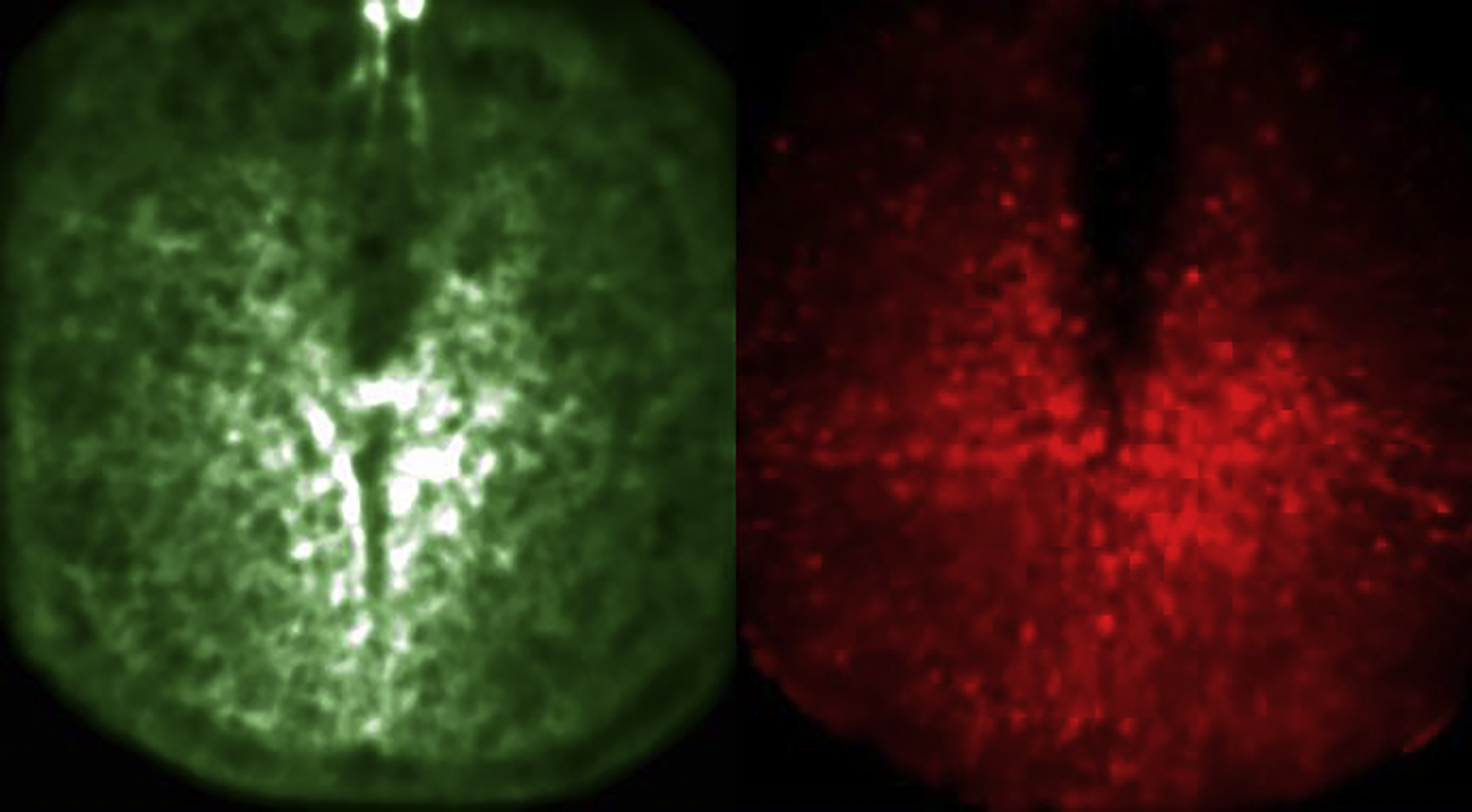

Supplement: Movie S1. Anti-phasic Rhythms of Neuronal [Ca2+]i and Extracellular [Glu]e in the Suprachiasmatic Nucleus — Related to Figure 1. Long-term fluorescent live imaging of SCN slices co-transduced with AAVs expressing the RCaMP1h or iGluSnFR to detect neuronal intracellular calcium and extracellular glutamate, respectively. Co-detection of these reporters revealed that sustained widespread anti-phasic oscillations [Ca2+]i and [Glu]e are present in the SCN. [file mmc2.jpg]

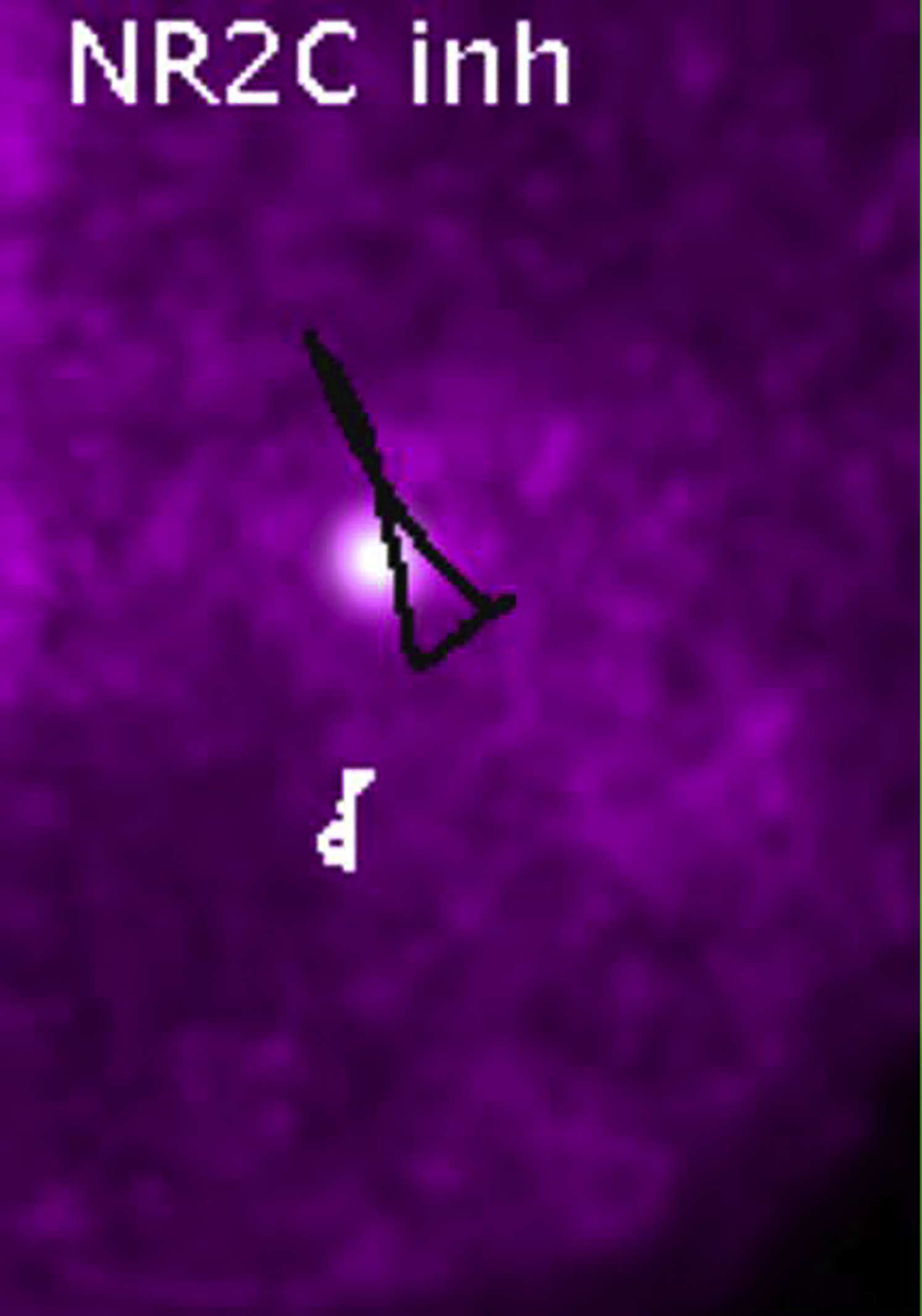

Supplement: Movie S2. CoL Analysis of PER2::LUC Spatiotemporal Wave in SCN Slices Treated with the NR2C Inhibitor DQP-1105 — Related to Figure 4. SCN slices expressing PER2::LUC were treated with the NR2C antagonist DQP-1105. Following drug addition, the original daily trajectory of the CoL of the bioluminescent distribution (black contour) shifted to a more ventral position (white contour), coherently with the dorsal expression of the NR2C subunit. [file mmc3.jpg]

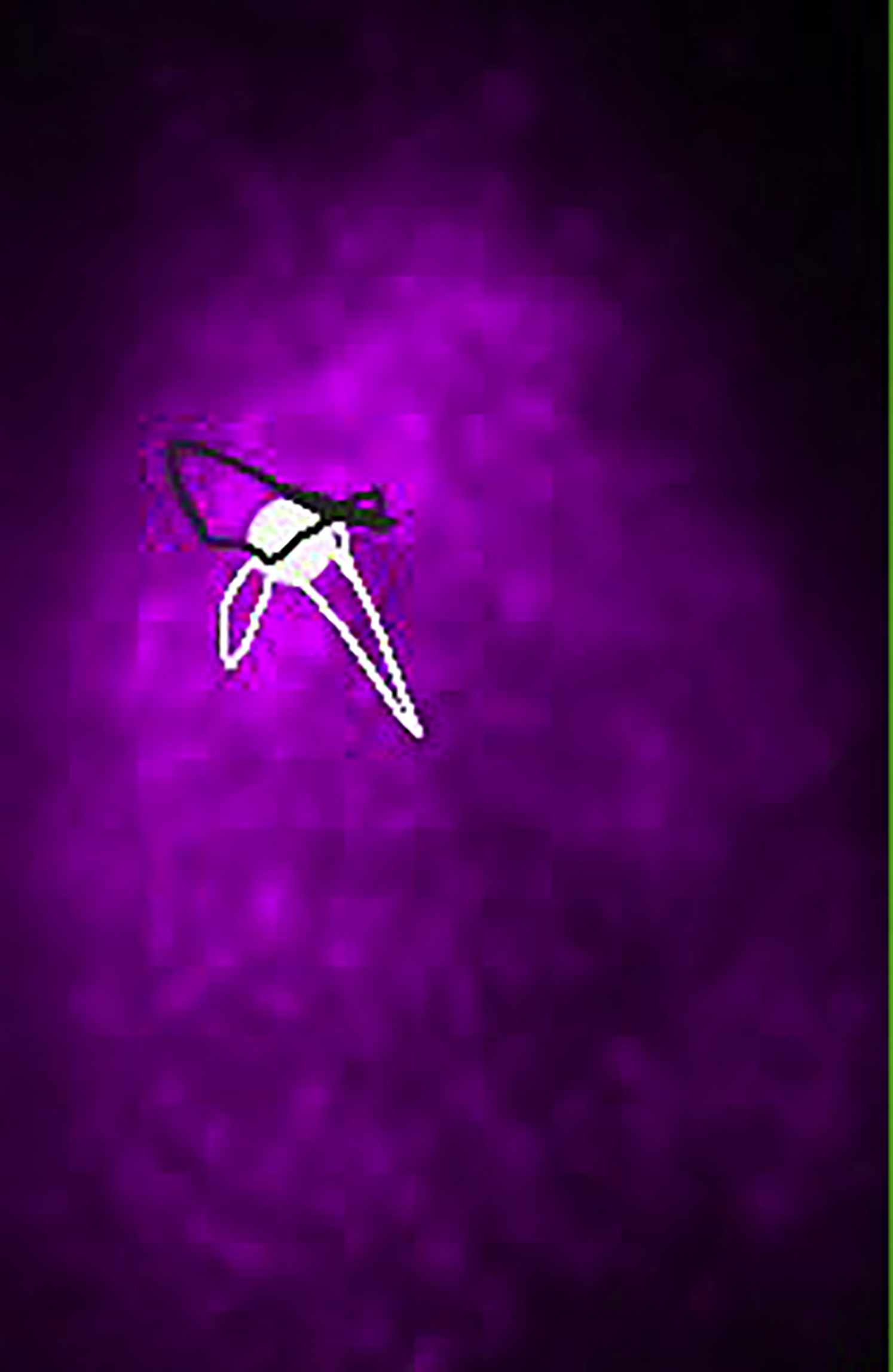

Supplement: Movie S3. CoL Analysis of PER2::LUC Spatiotemporal Wave in Astrocytically Restricted Ck1εTau/Tau Knockout — Related to Figure 7. SCN slices expressing PER2::LUC from floxed Ck1εTau/Tau mice were transduced with AAVs encoding Cre recombinase, driven by the GFAP promoter. Real-time bioluminescent recording started immediately after AAV transduction to follow the dynamic of the spatiotemporal changes in PER2::LUC expression upon astrocytic knockout of the Tau allele. After a lag, due to viral transduction cycle, the original daily trajectory of the CoL of the bioluminescent distribution (white contour) changed its shape and dynamically shifted to a more dorsal stable position (black contour). Therefore, re-programming of TTFL clock in astrocytes re-programmed the spatiotemporal wave of clock gene expression of the SCN to a new and specific state. [file mmc4.jpg]
